# Metabolite phosphatase from anhydrobiotic tardigrades

**DOI:** 10.1111/febs.17296

**Published:** 2024-10-17

**Authors:** Subaru Kato, Koki Deguchi, Masanori Obana, Yasushi Fujio, Yohta Fukuda, Tsuyoshi Inoue

**Affiliations:** ^1^ Graduate School of Pharmaceutical Sciences Osaka University Suita Japan; ^2^ Integrated Frontier Research for Medical Science Division, Institute for Open and Transdisciplinary Research Initiatives (OTRI) Osaka University Suita Japan

**Keywords:** phosphatase, stress tolerance, structural biology, tardigrade, X‐ray crystallography

## Abstract

Terrestrial organisms have systems to escape from desiccation stresses. For example, tardigrades (also known as water bears) can survive severe dried and other extreme environments by anhydrobiosis. Although their extraordinary ability has enchanted people, little is known about the detailed molecular mechanisms of anhydrobiosis. Here, we focused on the tardigrade *Ramazzottius varieornatus*, one of the toughest animals on Earth. A transcriptome database of *R*. *varieornatus* shows that genes encoding a Ferritin‐like protein are upregulated during desiccation or ultraviolet radiation. This protein shows sequence similarity to enigmatic proteins in desiccation‐tolerant bacteria and plants, which are hypothesized to be desiccation‐related. However, because these proteins lack detailed biological information, their functions are relatively unknown. We determined an atomic (1.05 Å) resolution crystal structure of a Ferritin‐like protein from *R*. *varieornatus*. The structure revealed a dinuclear metal binding site, and we showed that this Ferritin‐like protein has phosphatase activity toward several metabolite compounds including unusual nucleotide phosphates produced by oxidative or radiation damage. We also found that a homologous protein from a desiccation‐ and ultraviolet‐tolerant bacterium *Deinococcus radiodurans* is a metabolite phosphatase. Our results indicate that through cleaning up damaged metabolites or regulation of metabolite levels, this phosphatase family can contribute to stress tolerances. This study provides a clue to one of the universal molecular bases of desiccation‐stress tolerance.

## Graphical abstract

In this study, we determined the crystal structure of a Ferritin‐like protein widely distributed among intriguing organisms such as tardigrades, which show tolerance to extreme environmental conditions. We identified a dinuclear metal‐binding site, which we found harbors phosphatase activity toward several metabolites, including compounds related to cellular damage. Our findings provide an insight into one of the universal molecular bases of stress tolerance.

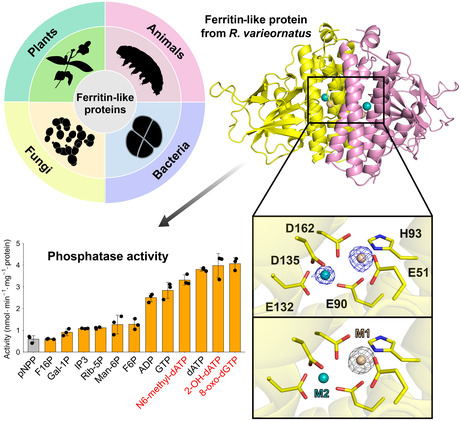

**Abbreviations**
BLAST: the basic local alignment search tool
CL: crossover linker
dNTP: deoxynucleotide triphosphate
ER: endoplasmic reticulum
FeMP: ferritin‐like metabolite phosphatase
GFP: green fluorescent protein
HGT: horizontal gene transfer
IgG: immunoglobulin G
MBP: maltose‐binding protein
*p*NPP: *p*‐nitrophenyl phosphate
RMSD: root mean square deviation
RNR: ribonucleotide reductase
*Rv*
: *Ramazzottius varieornatus*
Tar‐fer: tardigrade Ferritin‐like protein
UV‐C: ultraviolet C

## Introduction

Desiccation is an environmental stress to which terrestrial organisms are constantly subjected. Severe desiccation leads to loss of cytoplasmic water, destabilization of biological compounds, and suspension of metabolic activity, resulting in cell death in the worst case. During the evolution by which living systems expanded their habitats from the water to the land, they had to cope with severe water shortages caused by drought, high temperatures, and salinity [1]. Thus, terrestrial organisms have the systems to escape from the intense desiccation stress through regulation of genes and morphological changes of living tissues [2, 3].

While many unicellular organisms such as bacteria can endure dried conditions, complex multicellular organisms are usually more vulnerable to desiccation. However, some multicellular organisms such as nematodes, rotifers, and insects show high resistance to desiccation [4, 5]. One of the most desiccation‐tolerant organisms in the animal kingdom is the terrestrial tardigrade (also called water bears) [6, 7]. Tardigrades are microscopic invertebrates that inhabit water and terrestrial environments throughout the world [8]. Some tardigrades enter the state called anhydrobiosis and acquire desiccation tolerance [9]. To prepare anhydrobiosis, tardigrades shrink their bodies and undergo the morphological change to the “tun” state [10], suspending physiological processes and metabolic activities as if they are dead. Once desiccated tardigrades are rehydrated, they soon revert to the active state. In the anhydrobiotic state, tardigrades can survive not only desiccation but also other extreme environments such as low‐temperature [11], high‐temperature [12], high‐pressure [13], and radiation environments [14, 15]. These abilities are extraordinary among metazoans because tardigrades even have complex components including brain and nervous systems [16], which are easily damaged by severe environmental stresses. The understanding of their biological systems to survive extreme environments will serve a foundation of strategies for protecting organs, cells, and biomolecules as well as a partial answer to “what is life?”

Recently, biochemical and bioinformatic studies have revealed some clues about the extreme environmental tolerance of tardigrades. The transcriptome analyses from *Ramazzottius varieornatus*, one of the toughest tardigrades, and another anhydrobiotic tardigrade *Hypsibius exemplaris* revealed that expression of various genes fluctuate between active and anhydrobiotic states [17, 18], suggesting that many proteins are involved in protecting cells from dried environments. In fact, recent molecular analyses of *R. varieornatus* demonstrated the importance of tardigrade‐specific heat‐soluble protein families, such as secretory abundant heat‐soluble (SAHS) proteins [19], cytoplasmic abundant heat‐soluble (CAHS) proteins [19, 20], mitochondrial abundant heat‐soluble (MAHS) proteins [21], and damage suppressor (Dsup) protein [18, 22]. These proteins are conserved among Eutardigrada but not found in Heterotardigrada [23, 24]. Therefore, more ubiquitous molecules found across anhydrobiotic tardigrades have to be explored. More recently, a tardigrade‐specific protein family conserved both in Eutardigrada and Heterotardigrada have been identified. This protein family is proposed to function as a manganese peroxidase [25] or a calcium‐binding protein [26], but its cellular function is still unknown.

Other possible key factor involved in tardigrade anhydrobiosis is horizontal gene transfer (HGT) from other organisms although functions of HGT genes in tardigrade genomes have been controversial. A previous study claims that the genome of *H. exemplaris* contains more horizontally transferred genes compared to most animals and the elevated level of HGT may assist the anhydrobiotic ability [27]. Other independent studies object to this view, showing that *H*. *exemplaris* as well as *R. varieornatus* does not show elevated levels of HGT and foreign genes accounts for only 1–2% of whole genes [17, 18]; however, some HGT products such as bacteria‐derived catalases are conserved in tardigrades and appear to be related to stress tolerances [17, 18, 23].

In this study, we focused on structural genes of *R. varieornatus*, RvY_06210 and RvY_17634 (45.9% sequence identity to each other), which are annotated as products of HGT. Because they code Ferritin‐like proteins, we henceforward call them tardigrade Ferritin‐like proteins (Tar‐fers). A previous transcriptome data displays that the expression level of RvY_06210 increased in the anhydrobiotic state compared to that in the active state [18]. Another transcriptome data shows that RvY_17634 is upregulated when *R. varieornatus* is exposed to ultraviolet C (UV‐C) [25]. Tar‐fers are found not only in anhydrobiotic Eutardigrada such as *R. varieornatus* and *H*. *exemplaris* but also in anhydrobiotic Heterotardigrada such as *Echiniscus testudo* [25], suggesting that they contribute to extreme environmental tolerances in several tardigrade lineages. However, because they have not been biochemically characterized so far, their functions are totally unknown. Here, we explored the structure and function of Tar‐fers by X‐ray crystallography and enzymatic assays. As demonstrated below, Tar‐fers have an activity of metabolite phosphatase, so we hereafter call the RvY_06210 and RvY_17634 products *Rv*FeMP‐1 and *Rv*FeMP‐2 (*R. varieornatus* Ferritin‐like metabolite phosphatase 1 and 2), respectively.

## Results

### Amino acid sequences of Tar‐fers and related proteins

**Fig. 1:**
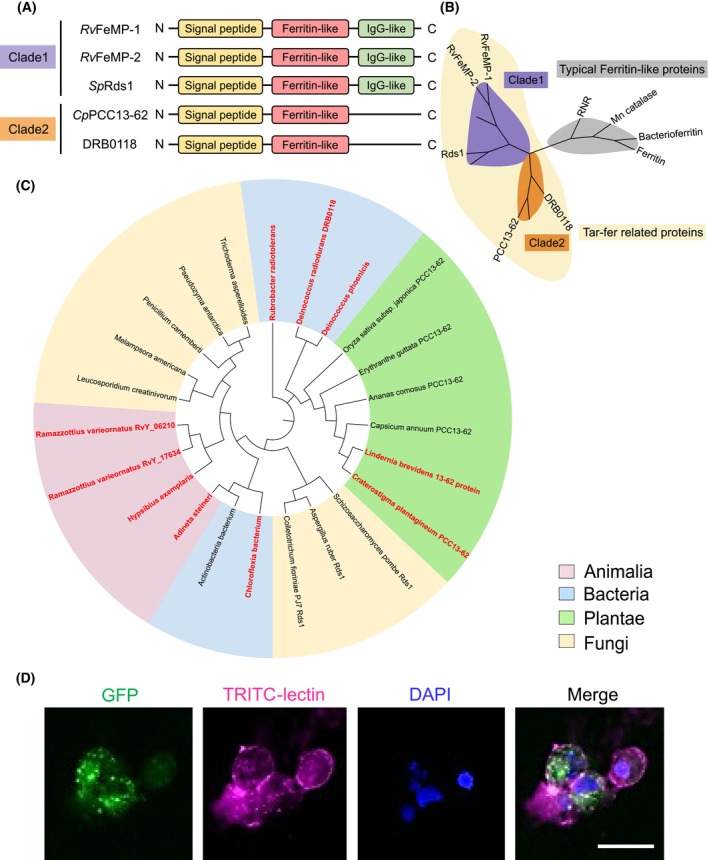
Ferritin‐like proteins widely conserved among various kingdoms of life. (A) Schematic view of domain configurations of Tar‐fers and representative Tar‐fer related proteins. They are divided into two clades depending on whether they have an IgG‐like domain or not. (B) Tar‐fers and related proteins are distinct from typical and well‐characterized Ferritin family proteins. The sequence similarities of Ferritin‐like domains between *Rv*FeMP‐1 and Tar‐fer related proteins are 17.6%, 19.6%, and 23.4% for *Cp*PCC13‐62, DRB0118 and *Sp*Rds1, respectively. On the other hand, the sequence similarities between *Rv*FeMP‐1 and known Ferritin‐like proteins are 12.7%, 13.4%, and 14.8% for human mitochondrial Ferritin, bacterioferritin from *Escherichia coli* and manganese catalase from *Lactobacillus plantarum*, respectively. Phylogenetic classification was performed by using Ferritin‐like domains. Representative genes in each clade are labeled. (C) Phylogenetic tree of various Tar‐fer related proteins generated by the maximum‐likelihood method using mega11. Names of organisms that are reported to have extreme environmental tolerances are depicted by red. The amino acid sequences used in this phylogenetic analysis are shown in Supporting Information. (D) Expression of GFP‐fusion *Rv*FeMP‐1 in HEK293T cells. Each cell was co‐stained by TRITC‐lectin (cell membrane) and DAPI (nucleus) after 48 h of transfection. The experiment was repeated twice with similar results. The scale bar indicates 25 μm.

Tar‐fers are predicted to be composed of a Ferritin‐like domain and an immunoglobulin G (IgG)‐like domain (Fig. 1A). The Ferritin‐like domain has a four‐helix bundle with a left‐handed twist and contains a dinuclear metal‐binding site. The IgG‐like domain is composed of two‐layer antiparallel β‐sheets, forming β‐barrel or sandwich structure. A default blast search using various Ferritin‐like proteins revealed that the Ferritin‐like domain of Tar‐fers showed low sequence similarity to canonical Ferritin‐like proteins such as ferritins [28], manganese catalases [29], and ribonucleotide reductases (RNRs) [30]. This result implies that Tar‐fers are phylogenetically classified into the subfamily different from well‐characterized Ferritin‐like proteins (Fig. 1B).

We then performed the blast search for other Ferritin‐like proteins, sequences of which show similarity to Tar‐fers. We found that homologous proteins (hereinafter called Tar‐fer related proteins) are distributed in a wide range of organisms including animals, fungi, plants, and bacteria (Fig. 1B,C). These uncharacterized proteins have been classified as a protein family named PFAM domain PF13668 or Ferritin_2 in the Pfam database.

It is noteworthy that some Tar‐fer‐related proteins are found in organisms displaying extraordinary stress tolerances (Fig. 1C red). An example is PCC13‐62, a protein product of a desiccation‐related gene, *pcC13‐62*. It was first discovered from a resurrection plant *Craterostigma plantagineum*, which is one of the most desiccation‐tolerant species in the plant kingdom [31]. The protein *Cp*PCC13‐62 (UniProt entry ID: P22242) contains an N‐terminal secretory signal peptide region like Tar‐fers but lacks an IgG‐like domain. Therefore, we classified it as a clade 2 Tar‐fer‐related protein (Fig. 1A,B). Although *Cp*PCC13‐62 has yet to be characterized at a molecular level, there are several reports that imply a relationship between the protein and desiccation tolerance: transcripts of *pcC13‐62* accumulate at high level in desiccation‐tolerant plants [32], and its expression is induced by desiccation, abscisic acid, and salt stress [33].

DRB0118 (UniProt entry ID: Q9RZK8) from *Deinococcus radiodurans* R1, a bacterium with phenomenal tolerance against desiccation and UV radiation [34], is also a homologous protein of Tar‐fers. The domain composition of DRB0118 is the same as *Cp*PCC13‐62 (Fig. 1A). In fact, DRB0118 was first reported as a Ferritin‐like protein similar to *Cp*PCC13‐62 [35]. Gene inactivation of DRB0118 reduces the survival rate of the bacterium under desiccation [36], indicating that it plays an important role in desiccation tolerance.

Tar‐fers also have amino acid sequence similarity to a product of *rds1* (named for “regulated by different signals”), a stress‐related gene in fungi. It was initially characterized as an adenine‐repressible gene in *Schizosaccharomyces pombe* and its expression level changes under glucose, ammonium, and phosphate starvation [34, 37]. However, molecular functions of the protein product Rds1 (UniProt entry ID: P53693) are still unknown.

Previous studies mentioned above implies that Tar‐fer‐related proteins have a relationship to various stress tolerances. However, because the family PF13668 is one of the Pfam entries with no experimentally determined structure and little biological information, their functions are unknown.

### Expression of *Rv*FeMP‐1 in eukaryotic cells

Subcellular localization of *Rv*FeMP‐1 was investigated by *Rv*FeMP‐1 fused with green fluorescent protein (GFP) and expressed in HEK293T cells. *Rv*FeMP‐1 and other Tar‐fer‐related proteins have the N‐terminal secretory signal peptide sequence, indicating that they are secretory proteins. However, an earlier study reported that Rds1 of *S. pombe* (*Sp*Rds1) localizes in the endoplasmic reticulum (ER) [38]. Our cellular experiments also showed that *Rv*FeMP‐1‐GFP was mainly distributed in cytoplasm (Fig. 1D) and *Rv*FeMP‐1 was not a secretory protein. At first, we hypothesized that *Rv*FeMP‐1 would localize in the ER as well as *Sp*Rds1. However, the ER marker (Bip/Grp78) and GFP signals of *Rv*FeMP‐1‐GFP did not completely coincide (Fig. S1). Since several strong GFP signals were observed in drop‐like structures, *Rv*FeMP‐1 may localize in certain cellular organelles or compartments. Our cellular experiments imply that *Rv*FeMP‐1 contributes to the interaction with intracellular molecules.

### Overall structure of *Rv*FeMP‐1

**Fig. 2:**
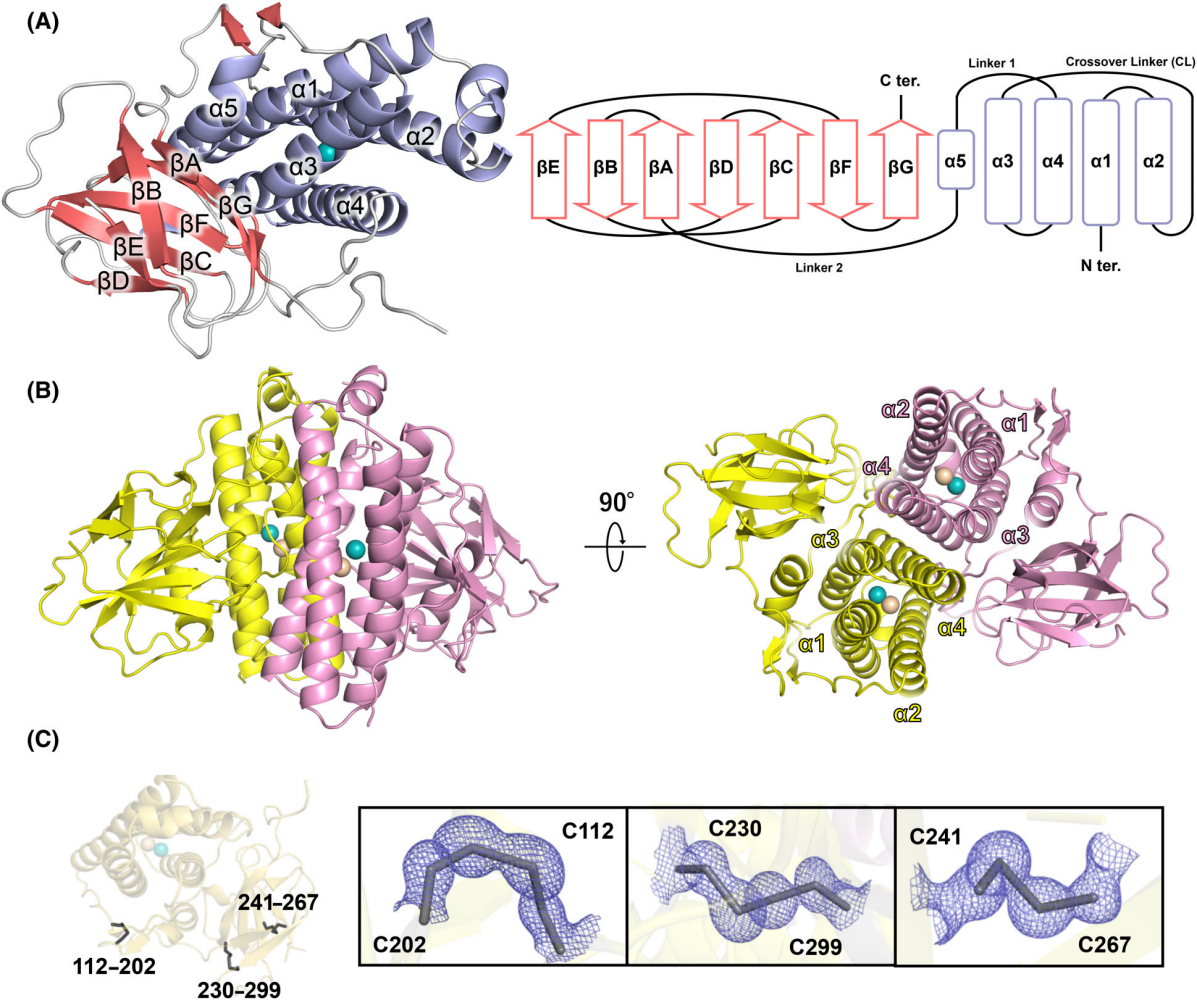
Overall crystal structure of *Rv*FeMP‐1. (A) Overall structure of *Rv*FeMP‐1 (left). Secondary α‐helices, β‐strands, and loops are colored in purple, pink and gray, respectively. Domain architecture of *Rv*FeMP‐1 (right). Ferritin‐like and IgG‐like domains consist of four α‐helices (purple) and seven β‐strands (pink). (B) Overall structure of *Rv*FeMP‐1. Each protomer is colored differently (yellow, pink) and shown by cartoon representation. Metal ions in the M1 and M2 sites are shown by cyan and wheat spheres, respectively. (C) Disulfide bonds in *Rv*FeMP‐1. A monomer of *Rv*FeMP‐1 is shown by cartoon representation. Three disulfide bonds (Cys112‐Cys202 [interdomain], Cys230‐Cys299, and Cys241‐Cys267 [intradomain]) are shown by black sticks. 2*mF*_o_–*DF*_c_ maps at 1.0 σ are shown by blue meshes.

We performed X‐ray crystallographic analysis of recombinant *Rv*FeMP‐1 produced in *Escherichia coli*. The crystal structure of *Rv*FeMP‐1 was determined at 1.05 Å resolution (Fig. 2A,B, Table 1). The asymmetric unit contained two *Rv*FeMP‐1 molecules with a Cα root mean square deviation (RMSD) value of 0.15 Å, suggesting that each molecule has almost identical conformations. The Ferritin‐like domain has a helix bundle composed of four α‐helices (α1–α4) and a crossover linker (CL) between α2 and α3, which are common features for the typical Ferritin‐like superfamily [39]. *Rv*FeMP‐1 also has a short helix (α5) at the end of the Ferritin‐like domain, which is similar to Ferritin and bacterioferritin that have a short helix at the C‐terminal region. The helix α5 is connected to α4 through a linker (Linker 1). The carbonyl oxygen atom of Pro181 on Linker 1 directly forms a hydrogen bond with a metal ligand (His93) of the neighboring molecule (Fig. S2).

**Table 1:** Data collection and refinement. Statistics for the highest‐resolution shell are shown in parentheses.

	*Rv*FeMP‐1 Zn/Mg	*Rv*FeMP‐1 Zn/Zn	*Rv*FeMP‐1 apo
Crystallization condition	B	A	C
Data collection			
Diffraction source	SPring‐8 BL44XU		
Wavelength (Å)	0.9		
Space group	*C*2	*P*2_1_2_1_2_1_	*P*2_1_
*a*, *b*, *c* (Å)	89.36, 64.02, 45.99	60.96, 82.16, 145.07	61.27, 80.81, 105.55
α, β, γ (°)	90.0, 109.54, 90.0	90.0, 90.0, 90.0	90.0, 91.26, 90.0
Resolution (Å)	37.00–1.05 (1.07–1.05)	48.96–1.45 (1.47–1.45)	44.60–1.70 (1.73–1.70)
Total reflections	371 381	866 575	319 041
Unique reflections	108 442 (5112)	129 382 (6276)	104 042 (5561)
Completeness (%)	95.8 (91.7)	99.8 (99.1)	97.3 (62.1)
Redundancy	3.4 (3.6)	6.7 (6.7)	3.1 (2.9)
*I*/σ (*I*)	14.9 (1.8)	16.1 (1.4)	9.1 (3.2)
CC_1/2_	0.998 (0.760)	0.999 (0.586)	0.973 (0.621)
*R*_merge_ (all I+ and I−)	0.039 (0.576)	0.059 (1.320)	0.100 (0.684)
*R*_meas_ (all I+ and I−)	0.046 (0.676)	0.064 (1.431)	0.122 (0.843)
*R*_pim_ (all I+ and I−)	0.025 (0.352)	0.025 (0.545)	0.068 (0.486)
Refinement			
Resolution (Å)	37.02–1.05 (1.09–1.05)	40.58–1.45 (1.50–1.45)	40.40–1.70 (1.76–1.70)
No. of reflections, working set	108 436 (10 411)	129 284 (12 695)	101 797 (11 243)
No. of reflections, test set	5426 (514)	6473 (645)	4990 (566)
*R*_work_/*R*_free_ (%)	12.86/15.45 (26.62/26.75)	16.78/19.22 (29.87/30.74)	18.42/22.76 (21.22/28.16)
RMSD bond length (Å)	0.006	0.006	0.007
RMSD bond angle (°)	0.89	0.85	0.86
Average *B*‐factor (Å^2^)			
Overall	19.3	24.6	19.7
Protein	17.7	22.2	18.7
Metal	8.19	31.3	–
Water	27.6	35.1	26.7
Ramachandran plot (%)			
Favored	97.74	98.73	98.15
Allowed	2.26	1.27	1.85
Outliers	0.00	0.00	0.00
PDB code ID	8KCE	8WAI	8W9K

The IgG‐like domain of *Rv*FeMP‐1 follows a linker region after α5 (Linker 2). Linker 2 interacts with CL and α5 through several hydrogen bonds and a disulfide bond between Cys112 and Cys202 (Fig. 2C, Fig. S2). The IgG‐like domain is composed of seven‐antiparallel β‐strands (βA to βG) to form a β‐sandwich structure. There are four cysteine residues in the IgG‐like domain, which form intradomain disulfide bonds (Fig. 2C). These disulfide bonds stabilize the structure and organize domain arrangement. There are almost no direct interactions between the Ferritin‐like domain and the IgG‐like domain. Functions of immunoglobulin superfamily are diverse and they are often involved in cell interactions and immune systems as recognition units for proteins and small molecules [40]. The function of the IgG‐like domain of Tar‐fers is currently unknown, but it may contribute to binding to other proteins or recognition of ligands.

**Fig. 3:**
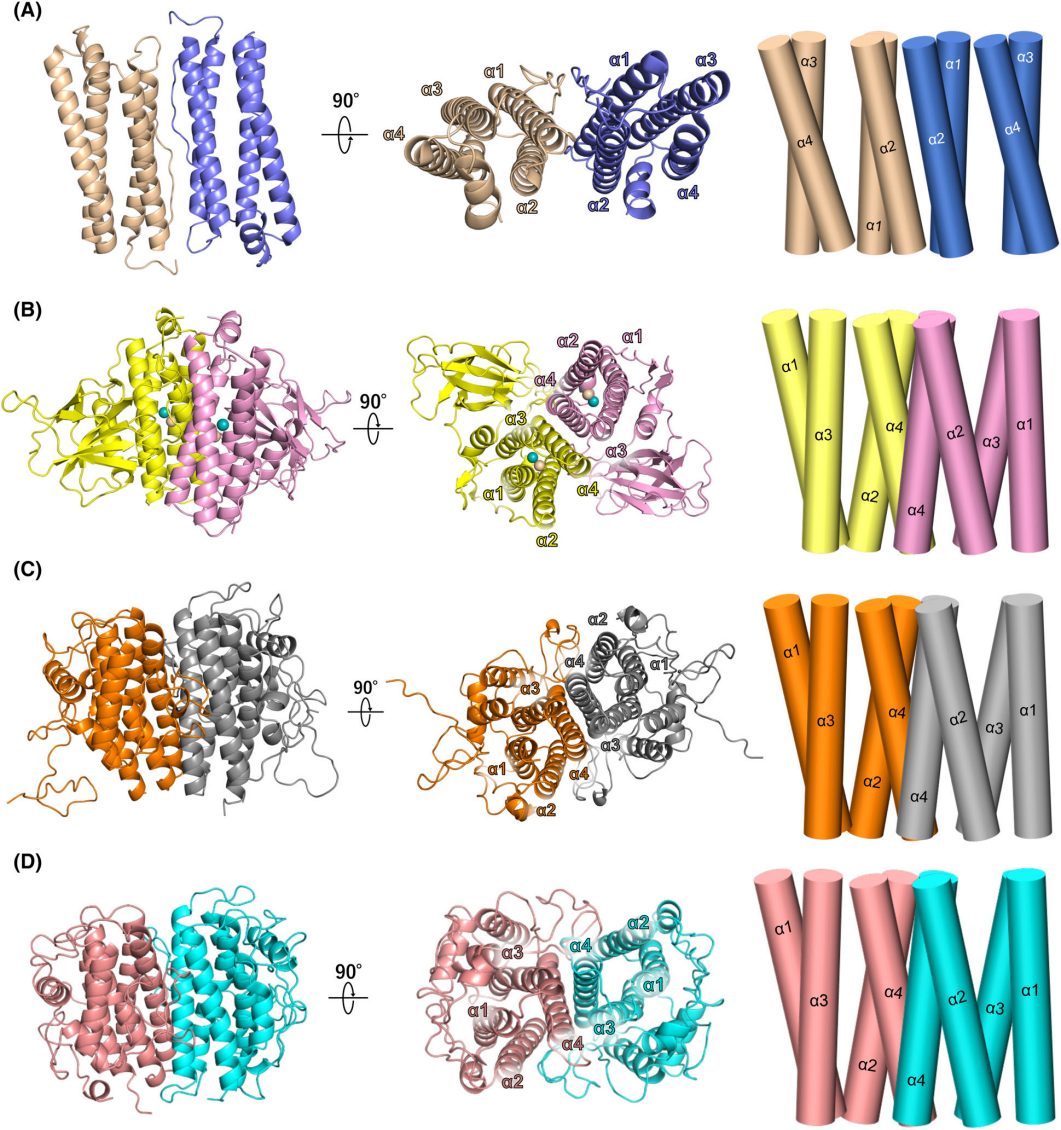
The difference of dimerization geometries. Cartoon representation for overall dimer structures of human mitochondrial ferritin (A) (PDB ID: 1R03), *Rv*FeMP‐1 (B), *Cp*PCC13‐62 (C) and DRB0118 (D). The model structures of *Cp*PCC13‐62 and DRB0118 were predicted by colabfold. The arrangement of α‐helices is shown in the cylinder diagram (right).

The structural topology of Ferritin‐like folds is diverse, including a simple fold composed of four helices and a complicated fold composed of more than 10 helices [39]. RvY_06210 has the simplest topology, consisting only of the four‐helix bundle, similar to Ferritins and bacterioferritins. Typical Ferritin‐like proteins form a dimetric structure that often builds up a larger complex such as 12‐meric and 24‐meric spherical structures, while others, such as RNRs, fatty acid desaturases, and rubrerythrins do not form the spherical structure [39]. Two *Rv*FeMP‐1 molecules in the asymmetric unit forms a dimer. The dimer interface of the well‐characterized Ferritin‐like proteins is composed of α1 and α2 (Fig. 3A) [39]. In contrast, protomers of the *Rv*FeMP‐1 dimer interacts with each other via α4 (Fig. 3B); that is, *Rv*FeMP‐1 forms the dimer with a topology distinct from those of known Ferritin‐like proteins. Using a purified sample of DRB0118, we tested if clade 2 Tar‐fer related proteins also form a dimer. The result of high resolution clear native PAGE revealed that it mainly forms a dimetric structure in solution (Fig. S3). We predicted dimerization structures of clade 2 Tar‐fer related proteins, *Cp*PCC13‐62 and DRB0118, with AlphaFold 2. Their dimerization geometry is the same as that of *Rv*FeMP‐1 (Fig. 3C,D), which further support that they are classified as Tar‐fer‐related proteins.

### Metal‐binding site

**Fig. 4:**
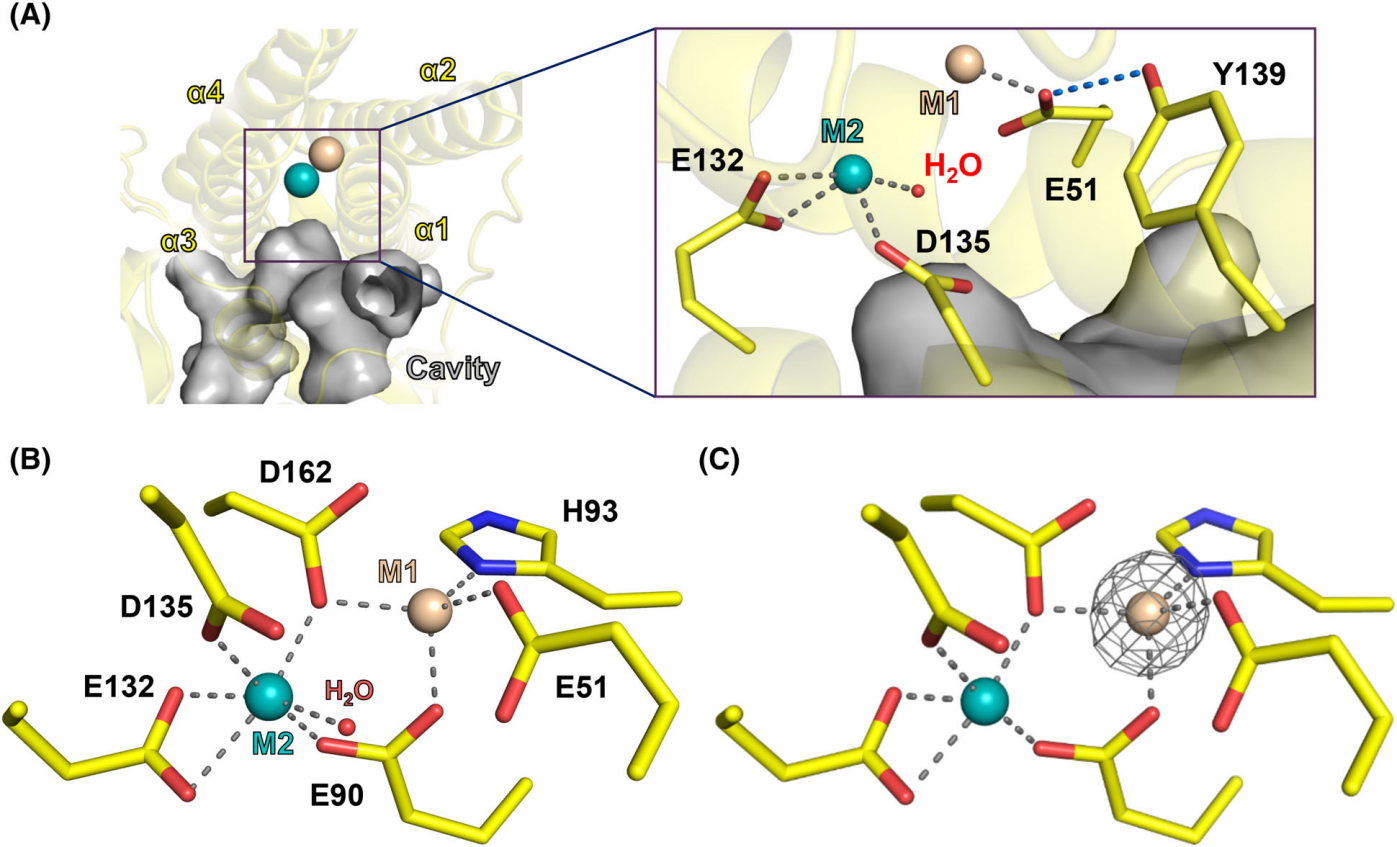
Metal‐binding site of *Rv*FeMP‐1. (A) Cavity of *Rv*FeMP‐1 toward the metal binding site. Metal ions in the M1 and M2 sites are shown by cyan and wheat spheres, respectively. The inset shows a close‐up view. Coordination and hydrogen bonds are shown by gray and blue dashed lines, respectively. A water molecule is represented by a small red sphere. (B) Close‐up view for the metal binding site of *Rv*FeMP‐1. (C) Anomalous scattering signals from the Zn‐Mg *Rv*FeMP‐1 data. Anomalous peaks are shown as gray meshes contoured at 10 σ. The wavelength of X‐ray to collect this data was 1.28 Å.

The structure of *Rv*FeMP‐1 shows a cavity that leads from the molecular surface to the center of the α‐helix bundle (Fig. 4A). There is a dinuclear metal center (M1 and M2 in Fig. 4B) at the bottom of the cavity. Anomalous scattering data confirmed the presence of one zinc ion (Zn^2+^) at the M1 site (Fig. 4C, Fig. S4A, Table S1). Because the other metal site (M2) did not show an anomalous peak, we modeled it as a light metal ion. The atomic resolution structure enabled us to know precise coordination distances between the amino acid ligands and the light metal ion (2.0 and 2.1 Å to Glu90 and Glu132, respectively), which corresponds to the typical coordination distance of Mg^2+^ [41]. Zn^2+^ ion (M1) is coordinated by Glu51, Glu90, His93, and Asp162, which are residues highly conserved among the metal binding sites of the Ferritin‐like superfamily [42]. Mg^2+^ ion (M2) is stabilized by coordination of Glu90, Glu132, Asp135, Asp162, and a water molecule. All four helices of the Ferritin‐like domain are involved in coordination to metal ions. The majority of the Ferritin‐like superfamily have a homo‐dinuclear heavy metal binding site and only a few, such as RNRs, have a hetero‐dinuclear one [30]. Our crystal structure of *Rv*FeMP‐1 shows a Zn‐Mg hetero‐dinuclear metal‐binding site, unlike common Ferritin‐like proteins. It could be an artifact due to replacement of Zn^2+^ at the M2 site with Mg^2+^ contained in the crystallization solution. In fact, anomalous scattering data from a crystal obtained in a different crystallization condition shows the presence of two Zn^2+^ ion at the metal center (Fig. S4B, Table 1, Table S1); however, the anomalous peak of the M2 site is about three times weaker than that of M1. Even though, we produce *Rv*FeMP‐1 in a zinc‐rich medium, the resulting sample contains Zn^2+^ at the M1 site but the M2 site shows low or negligible occupancy of Zn^2+^ (Fig. S4C, Table S1). These observations indicate that the M2 site is occupied by or easily replaced with other metal ions such as Mg^2+^. The crystal structure of known Ferritin‐like proteins, such as Ferritin from *Pyrococcus furiosus* (*Pf*Ftn), revealed that the occupancy of the M2 site is < 70%, unlike the M1 site [43]. Therefore, that a metal ion at the M2 site in *Rv*FeMP‐1 is easily desorbed and replaced is attributed to the common feature of Ferritin‐like proteins: lability of the M2 site. Our crystallographic experiments did not determine the true active metal state of *Rv*FeMP‐1.

We have also obtained the apo *Rv*FeMP‐1 structure from another crystal condition (Fig. S5, Table 1). Because the preparation condition of this crystal was acidic (pH 4.0), protonated amino acids around the active site would expel metal ions. By the superimposition between apo *Rv*FeMP‐1 and Zn‐Mg *Rv*FeMP‐1 (Fig. S5), we found that only Asp135 at the M2 site is a flexible residue which changes the orientation by metal binding, while the other residues remain almost unchanged. In bacterioferritin, the coordinating histidine residue at the M2 site, which is highly conserved among bacterioferritins, can assume different conformations [44]. This conformational change of the histidine ligand is proposed to be involved in Fe access to the ferroxidase center [45]. The conformational change of Asp135 may have a similar metal‐guiding role in *Rv*FeMP‐1.

### Structure comparison with other Ferritin‐like proteins

**Fig. 5:**
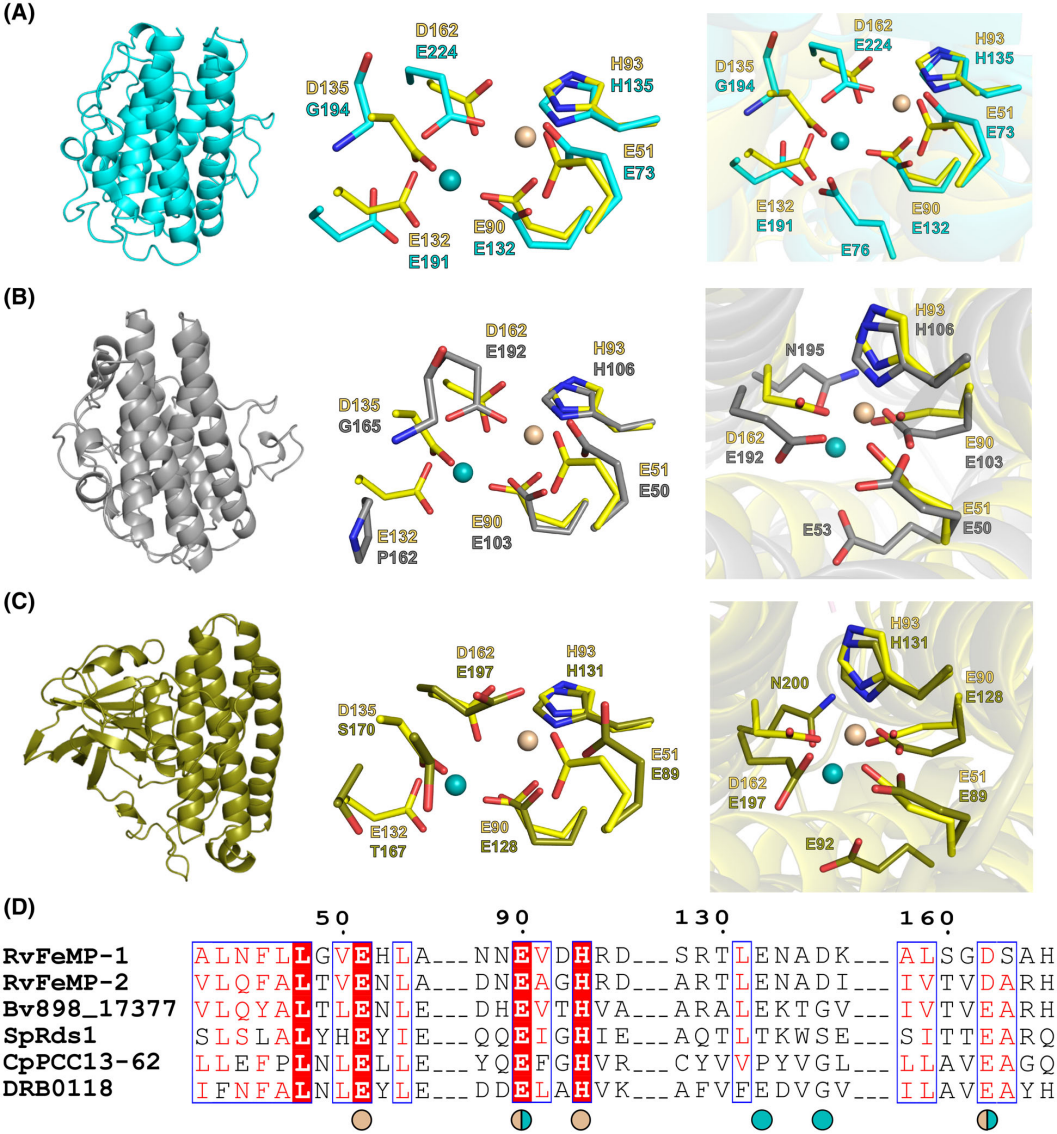
Structural comparison between *Rv*FeMP‐1 and its analogous proteins. The overall structures of (A) DRB0118 (cyan), (B) *Cp*PCC13‐62 (gray), and (C) *Sp*Rds1 (deep olive) were shown by cartoon in left. The superimpositions of metal sites of *Rv*FeMP‐1 (yellow) and each protein were shown by sticks in the middle and right. (D) Sequence alignment of *Rv*FeMP‐1 along with its analogous proteins by clustal omega. Wheat circles: residues which coordinate to M1. Cyan circles: Residues which coordinate to M2. Wheat/cyan circles: residues which coordinate to both M1 and M2. *Rv*FeMP‐1 (UniProt entry ID: A0A1D1V463), *Rv*FeMP‐2 (UniProt entry ID: A0A1D1W2V1), Bv898_17377 (UniProt entry ID: A0A9X6NEZ0): Tar‐fer of *Hypsibius exemplaris*, *Sp*Rds1 (UniProt entry ID: P53693): Rds1 of *Schizosaccharomyces pombe*, *Cp*PCC13‐62 (UniProt entry ID: P22242): PCC13‐62 in *Craterostigma plantagineum*. DRB0118 (UniProt entry ID: Q9RZK8).

The structure of *Rv*FeMP‐1 was superimposed with structures of DRB0118, *Cp*PCC13‐62, and *Sp*Rds1 predicted by AlphaFold2 [46] (Fig. 5A–C). Although overall amino acid sequence similarities between *Rv*FeMP‐1 and other Tar‐fer‐related proteins are not so high (23.8%, 19.5%, and 20.1% for DRB0118, *Cp*PCC13‐62, and *Sp*Rds1, respectively), residues of the M1 site (Glu51, Glu90, and His93 in *Rv*FeMP‐1) are conserved among Tar‐fer related proteins (Fig. 5D). Contrary to the M1 site, residues at the M2 site varies among Tar‐fer‐related proteins. While residues at Asp162 and Glu51 of *Rv*FeMP‐1 are Asp or Glu in other Tar‐fer‐related proteins, Asp135 on α3 in *Rv*FeMP‐1 is replaced with Gly194 in DRB0118, Gly165 in *Cp*PCC13‐62, and Ser170 in *Sp*Rds1. This lack of the ligand may be compensated by Glu76 in DRB0118, Glu53 in *Cp*PCC13‐62, and Glu92 in *Sp*Rds1 located on α1. Glu132 in *Rv*FeMP‐1 is conserved in DRB0118 (Glu191), but it is replaced by Pro162 in *Cp*PCC13‐62 and Thr167 in *Sp*Rds1. Because the side chain of Gln195 in *Cp*PCC13‐62 and Gln200 in *Sp*Rds1 could be located < 3.5 Å from the M2 atom, they may still coordinate the metal ion (Fig. 5B,C). In summary, structural comparison shows similarity of the metal center between Tar‐fer‐related proteins.

### Phosphatase activity

**Fig. 6:**
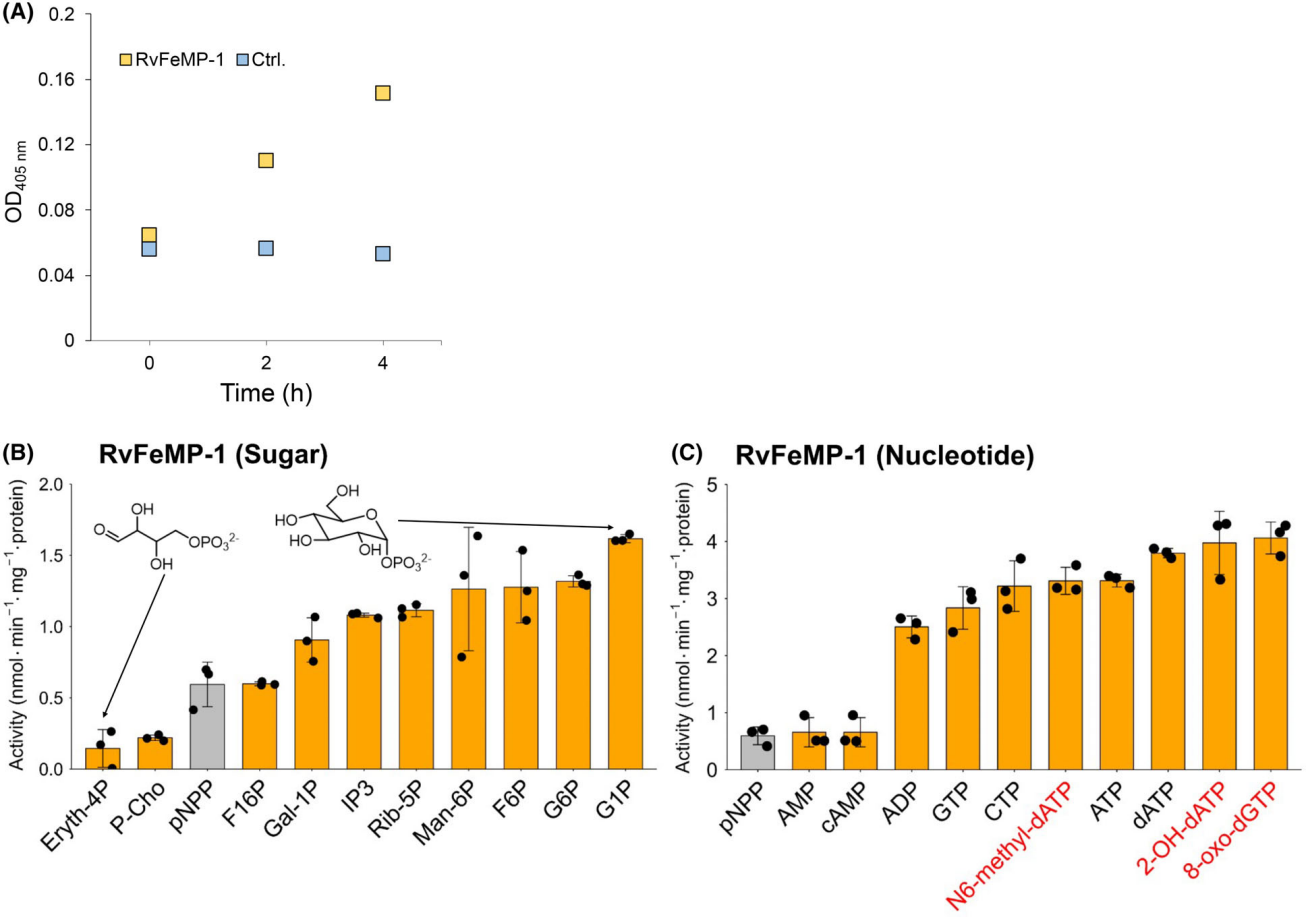
Enzymatic activity profiles of *Rv*FeMP‐1. (A) Phosphatase assay of *Rv*FeMP‐1 by using *p*NPP. The plot shows the change of absorbance at 405 nm. (B) Substrate scope of *Rv*FeMP‐1 toward sugar phosphates and P‐Cho. Chemical structures of glucose‐6‐phosphate (G6P) and erythrose 4‐phosphate (Eryth‐4P) are shown for comparison. (C) Substrate scope of *Rv*FeMP‐1 toward nucleotides. In (B, C), the artificial substrate *p*NPP is shown by gray bars. Data are mean ± SD of three technical replicates for all assays. Substrates shown by red characters are damaged nucleotides. Abbreviations of compounds are listed in Materials and methods section.

We predicted that *Rv*FeMP‐1 is an enzyme because it has the cavity that is wide enough to accommodate a small compound (Fig. 4A). Because some hydrolases are known to use the dinuclear metal site containing Zn^2+^ and/or Mg^2+^ [47], we expected that Tar‐fers would have a similar activity. When we examined an activity using an artificial substrate *p*‐nitrophenyl phosphate (*p*NPP), *Rv*FeMP‐1 showed a dephosphorylation activity (Fig. 6A).

**Fig. 7:**
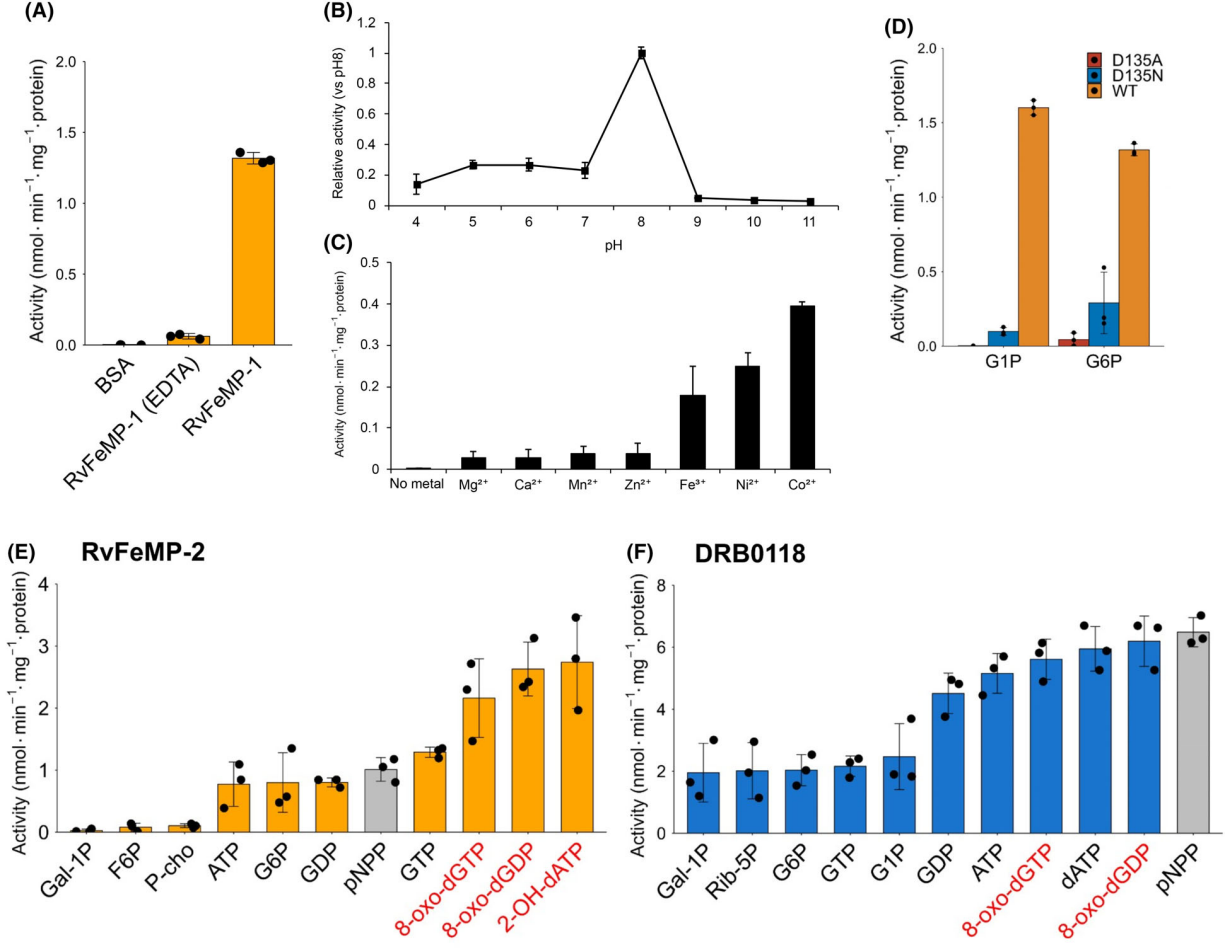
Enzymatic activity profiles of Tar‐fer and related proteins. (A) Enzymatic assay of *Rv*FeMP‐1. The condition with bovine serum albumin (BSA) but without *Rv*FeMP‐1 is a negative control, showing that the presence of protein molecules itself does not show the phosphatase activity. The presence of EDTA lowers the activity of *Rv*FeMP‐1. G6P was chosen as the substrate due to high activity. (B) pH assay of *Rv*FeMP‐1 using G6P as a substrate. Data are mean ± SD of three technical replicates for each pH condition. (C) Metal dependence of phosphatase activity. The assay was performed using the mixture with 10 μg apo *Rv*FeMP‐1, 5 mm G6P (substrate), 5 mm metal solution and 20 mm Tris–HCl pH 8.0 in a total volume of 50 μL. The apo (metal‐free) sample was produced by exposure to 5 mm EDTA solution overnight before purification by a SEC column. Other conditions are the same as mentioned above. Data are mean ± SD of three replicates. (D) Measurement of phosphatase activity by using D135A or D135N *Rv*FeMP‐1 mutants. G6P and G1P were chosen as substrates. Substrate scope of (E) *Rv*FeMP‐2 and (F) DRB0118 toward various substrates. In (E, F), the artificial substrate *p*NPP is shown by gray bars. Data are mean ± SD of three technical replicates for all assays. Substrates shown by red characters are damaged nucleotides. Abbreviations of compounds are listed in Materials and methods section.

To find more reactive substrates, we performed substrate screening for *Rv*FeMP‐1 using various phosphorylated compounds. *Rv*FeMP‐1 had higher activity toward several compounds than *p*NPP, in which sugar phosphates (Fig. 6B) and nucleotides (Fig. 6C) are included. Among sugar phosphates tested, glucose‐6‐phosphate (G6P) and glucose‐1‐phosphate (G1P) with a six‐membered ring structure are the best substrates for *Rv*FeMP‐1. On the other hand, *Rv*FeMP‐1 had lower activity toward a linear sugar phosphate erythrose 4‐phosphate (Eryth‐4P) as well as phosphocholine (P‐cho). The presence of a metal chelating reagent ethylenediaminetetraacetic acid (EDTA) decreases the activity of *Rv*FeMP‐1, confirming that this enzyme is metal dependent (Fig. 7A). The maximum activity was observed at pH 8 and the enzyme shows very low activity under acidic conditions (Fig. 7B), which is consistent with the empty metal‐binding site observed in the crystal structure at pH 4.0.

The assay of metal dependence was performed by using the EDTA‐treated metal‐free sample and revealed that phosphatase activity of *Rv*FeMP‐1 with Ni^2+^ and Co^2+^ was 5–8 times higher than with Zn^2+^ (Fig. 7C); however, the activity of the EDTA‐treated sample with Zn^2+^ was more than two orders of magnitude less active compared to that of the sample before EDTA treatment. This result implies that *in vitro* reconstruction of the metal center of *Rv*FeMP‐1 is difficult. As the result, we could not determine the true physiological composition of the metal site although Ni^2+^ and Co^2+^ may play an important role as physiological metal ions. Besides, introducing mutations at the metal center (D135A or D135N) abolished the phosphatase activity (Fig. 7D), showing that the metal site is the catalytic center and that Asp135 is necessary for the dephosphorylation reaction of *Rv*FeMP‐1.

*Rv*FeMP‐1 catalyzes dephosphorylation of a wide range of nucleotides regardless of differences in base structures or the number of phosphates (Fig. 6C). It is noteworthy that *Rv*FeMP‐1 can dephosphorylate several nucleotide phosphates with bases produced by oxidative or radiation damages (8‐oxo‐2′‐deoxyguanosine 5′‐triphosphate [8‐oxo‐dGTP], N6‐Methyl‐2′‐deoxyadenosine‐5′‐triphosphate [N6‐methyl‐dATP], and 2‐Hydroxy‐2′‐deoxyadenosine‐5′‐triphosphate [2‐OH‐dATP]).

In addition, we tested generality of our finding by using *Rv*FeMP‐2 and DRB0118 produced in and purified from *E. coli*. *Rv*FeMP‐2 showed the phosphatase activity and had the higher activity toward G6P and GTP than toward P‐cho, which is similar to the substrate specificity of *Rv*FeMP‐1 (Fig. 7E). DRB0118, a clade 2 Tar‐fer‐related protein, can also function as a phosphatase and the activity toward *p*NPP was higher than that of Tar‐fers (Fig. 7F). As was observed for *Rv*FeMP‐1, both *Rv*FeMP‐2 and DRB0118 had higher activity toward several nucleotides including damaged compounds.

## Discussion

In this study, we determined the crystal structure of the previously uncharacterized Ferritin‐like protein *Rv*FeMP‐1, which provided a clue to the biochemical function of Tar‐fer. Ferritin‐like family proteins have similar scaffolds but show various roles ranging from iron storage (Ferritin) [28] to reactive oxygen species scavenger (manganese catalase) [29] and synthesis of deoxyribonucleotide (RNR) [30]. Tar‐fers added further functional diversity to this family. It accommodates zinc or magnesium ions at the dinuclear site and dephosphorylates several metabolite compounds. Furthermore, our proposed reaction mechanism of dephosphorylation implied that a water molecule coordinated by the M2 site would attack a phosphate group of a substrate (Fig. S6A). All proteins tested in this study can react with a wide range of compounds. Their enzymatic activity profiles vary probably because they show differences of residue sets constituting the catalytic sites.

The phosphatase activities of examined proteins are 1–2 orders of magnitude lower than those of several known metabolite phosphatases [48]. The possible reason for this low activity is that we used recombinant samples that contain the enzymes with low metal occupancy and/or without posttranslational modification. Eukaryotic proteins expressed in *E*. *coli* often show lower activity. Although DRB0118 is a prokaryotic protein, we expressed it as a fusion protein with maltose‐binding protein (MBP) otherwise DRB0118 was unstable. The MBP‐tag may inhibit the activity of DRB0118 because it can provide a non‐native conformation and intercept substrates.

Accumulation of damaged metabolites generated by environmental stresses and promiscuous enzymatic reactions has devastating influences on cells [49, 50]. These damaged metabolites should be released into extracellular regions or removed by hydrolysis reactions of various house‐cleaning enzymes [50]. For instance, *E*. *coli* MutT [51] prevents the misincorporation of oxidized nucleotide 8‐oxoguanine into the genome by hydrolysis of 8‐oxo‐dGTP produced by reactive oxygen species or radiation. Human MutT homolog 1 (MTH1) [52] hydrolyzes another damaged nucleotide N6‐methyl‐dATP. These protein families stabilize cellular metabolism by sanitizing the deoxynucleotide triphosphate (dNTP) pools.

Some other phosphatases control cellular levels of normal metabolites by degrading the excess amounts of them. This function is called a “directed overflow” mechanism to maintain cellular homeostasis [53]. For example, *E*. *coli* acid glucose‐1‐phosphatase plays a crucial role in scavenging glucose, which is an important intermediate of glycogen biosynthesis [54]. Deoxynucleotide triphosphohydrolases regulates the amount of dNTP in cellular environments [55, 56], which contributes to the proper replication and maintenance of genome information.

Our study revealed that Tar‐fer‐related proteins that are distributed in various organisms can catalyze dephosphorylation of some phosphate compounds. Given that damaged metabolites are also included in substrates for Tar‐fer‐related proteins, these proteins would have functions to remove damaged compounds from intracellular regions. Besides, Tar‐fer‐related proteins dephosphorylate normal metabolites such as sugar phosphates. This indicated that Tar‐fer‐related proteins can also degrade the normal compounds to inhibit excessive condition, contributing to the “directed overflow” mechanism. Therefore, this protein family may be involved both in “removal of damaged metabolites” and “degradation of excessive non‐toxic metabolites” (Fig. S6B). Such proper management of metabolite pools will lead to the survival in harsh environments where damaged compounds accumulate and homeostasis balance is easily disrupted. While *Rv*FeMP‐1 is upregulated under desiccation, *Rv*FeMP‐2 by UV‐C irradiation. Considering that cells exposed to desiccation or UV‐C encounter similar problems such as damages to nucleotides and disorder of metabolic pathways, they must have similar physiological functions in the cell. Because *Rv*FeMP‐2 seems to distinguish damaged nucleotides from normal ones more clearly than *Rv*FeMP‐1 (Figs 6C and 7E), it may be especially upregulated by UV irradiation that rapidly generates damaged nucleotides. However, how these proteins are properly regulated and used depending on the situations is currently unknown.

Studies of molecular bases of anhydrobiosis are expected to lead to applications for protecting cells [18] and biologics [57] as well as for survival in space [7, 58]. Besides, mechanisms to surmount desiccation stresses have been vigorously investigated in the field of plant science because they will be the foundation for protection of crops from drought caused by the global climate change [1]. Unfortunately, a unique mechanism employed in one organism often cannot be used in another because each organism has evolved its own molecular systems to suite itself. Exploring universal mechanisms which can work in various cell systems is therefore important for technological advances. Because the enzyme is distributed across the kingdoms of life, our study provides not only insights into tardigrade anhydrobiosis but also a ubiquitous molecular basis in desiccation‐tolerant organisms. At the same time, our research leaves many questions unsolved, such as: what is the true substrate of Tar‐fers and related proteins? What are their physiological functions in cells? These mysteries have to be unlocked through interdisciplinary research; thus, our study will accelerate future studies in the broad fields. Moreover, our study indicates that some horizontally transferred genes in tardigrades are important for their biology although the extent to which HGT is responsible for tardigrade anhydrobiosis has been a matter of debate. Further analyses of horizontally transferred genes in anhydrobiotic tardigrades will lead to the deeper understanding of universal biological mechanisms of stress tolerances.

## Materials and methods

### Databases and sequence analyses

Protein sequences of interest were obtained from the database of The National Center for Biotechnology Information (https://www.ncbi.nlm.nih.gov). Amino acid sequence analyses were performed by mega11 [59]. blast searches were performed via the web service for protein blast (https://blast.ncbi.nlm.nih.gov/Blast.cgi) [60]. Phylogenetic analysis of the Ferritin‐like superfamily was performed by using only Ferritin‐like domains. A maximum likelihood method was used to estimate phylogenetic trees. Figures of phylogenetic trees were illustrated by using itol [61]. A figure of sequence alignment was generated by espript 3 (http://espript.ibcp.fr) [62]. Model structures predicted by AlphaFold2 (as of Aug 2023) were obtained from AlphaFold Protein Structure Database (https://alphafold.ebi.ac.uk/). The dimetric models of Tar‐fer related proteins were generated by using colabfold with mmseqs2 [63].

### Cloning of *Rv*FeMP‐1 and *Rv*FeMP‐2

*Ramazzottius varieornatus* strain YOKOZUNA‐1 was a kind gift from T. Kunieda at The University of Tokyo. It was reared on water‐layered agar plates by feeding Chlorella CHIKUGO (CK‐5) (Chlorella Industry Co., Ltd., Minato‐ku, Tokyo, Japan) at 22 °C as described previously [7]. Total messenger RNA (mRNA) was extracted from about 10 individuals of *R. varieornatus* strain YOKOZUNA‐1 in the active state with a Direct‐zol RNA Microprep kit (Zymo Research, Irvine, CA, USA) according to an instruction manual. A complementary DNA (cDNA) library was made by reverse transcription of total mRNA with a PrimeScript II 1st strand cDNA Synthesis kit (Takara Bio, Kusatsu, Shiga, Japan) and oligo dT primers. DNA fragments corresponding to genes of *Rv*FeMP‐1 (UniProt entry ID: A0A1D1V463) and *Rv*FeMP‐2 (UniProt entry ID: A0A1D1W2V1) were amplified by the polymerase chain reaction (PCR) with primers described in Table S2. The obtained fragments were inserted into a pET28a vector (Novagen, Madison, WI, USA) or a pAcGFP1‐N1 vector (Clontech, Mountain View, CA, USA) as described below. All DNA sequences were checked by DNA sequencing (Fasmac Co., Ltd., Atsugi, Kanagawa, Japan).

### Expression and purification of Tar‐fer

The expression construct of *Rv*FeMP‐1 contains an N‐terminal 6 × His tag followed by a TEV protease recognition site. SignalP 5.0, which predicts the location of signal peptide, revealed that 20 amino acids from the N‐terminus would be the secretory signal peptide sequence. Therefore, *Rv*FeMP‐1 was expressed excluding this region. The cDNA of *Rv*FeMP‐1 (21–316) was cloned into pET28a vector by an in‐fusion technique (Takara Bio). The final amino acid sequence is the following, in which italic characters indicate the *Rv*FeMP‐1 region and ENLYFQG is a TEV protease recognition site.

MGSSHHHHHHENLYFQG*GGNRGMSRNQDAYAEKMDMTLDALNFLLGVEHLASAFYVQAVNNFTADDFKAAGLAQRDYDQFVGVRNNEVDHRDTLISVIKSLGGKPNPPCKYTFPVTDVASVLKVSRTLENADKPAYLGALRDIKSVELRTSVQGALSGDSAHAAFFAYLTGKAPAPGPVDGPLTQRHIATLAQDFIVSCPYPAPKPFPKLTLSPQSGPVGTVVATTCAQDVDTNGVMCAIISGNQGTLMQRPGQAKDGSGAATCTIPPGVKGILFIAWVRGRDVLNVGVDDSSTVCGPNYFLLSALGDAVPGV*

Site‐directed mutagenesis, in which Asp135 was replaced with Ala (D135A) and Asn (D135N), was performed by PCR using the pET28a‐*Rv*FeMP‐1 plasmid and mutagenesis oligonucleotide primers shown in Table S2. The mutation points were checked by DNA sequencing. The pET28a‐*Rv*FeMP‐1 expression construct was transformed into *E. coli* Shuffle T7 (New England BioLabs, Ipswich, MA, USA). Transformed *E. coli* cells were grown at 37 °C in LB medium (Merck [Sigma‐Aldrich], Darmstadt, Germany) with 50 μg·mL^−1^ kanamycin sulfate (FUJIFILM Wako Pure Chemical Corporation, Osaka, Japan) until OD_600_ became 0.6–0.8. The protein expression was induced by the addition of isopropyl‐1‐thio‐β‐d‐galactopyranoside (IPTG) (Nacalai Tesque, Nakagyo‐ku, Kyoto, Japan) at a final concentration of 1 mm. After 20 h at 18 °C, cells were collected by centrifugation (6000 ***g***, 30 min) at 4 °C and resuspended in Buffer A (20 mm Tris–HCl pH 8.0, 300 mm NaCl, and 5% v/v Glycerol). Cells were lysed by sonication on ice, and cell debris was removed by centrifugation (20 000 ***g***, 60 min) at 4 °C. The supernatant was loaded onto a HiTrap TALON column (GE Healthcare, Chicago, IL, USA) previously equilibrated with buffer B (20 mm Tris–HCl pH 8.0, 200 mm NaCl, 5% v/v glycerol, and 5 mm imidazole). The column was then washed by Buffer B and His‐*Rv*FeMP‐1 was eluted by Buffer C (20 mm Tris–HCl pH 8.0, 200 mm NaCl, 5% v/v glycerol, and 200 mm imidazole). His‐*Rv*FeMP‐1 was mixed with TEV protease, and 6‐His tag was removed by the dialysis against 20 mm Tris–HCl pH 8.0 overnight at 4 °C. The sample was then filtered by a 0.45 μm membrane syringe filter to remove precipitation and loaded onto HisTrap column (GE Healthcare) previously equilibrated with buffer B. The flowthrough fraction was concentrated by a 10 kDa molecular weight cutoff Vivaspin tube (Sartorius, Goettingen, Hessen, Germany) and loaded onto a Superdex 200 Increase 10/300 size exclusion chromatography (SEC) column (GE Healthcare) equilibrated with SEC buffer (20 mm Tris–HCl pH 8.0). *Rv*FeMP‐1 fraction was concentrated to 10 mg·mL^−1^ (estimated from absorption at 280 nm and the extinction coefficient) and stored at −80 °C. The result of SEC purification is shown in Fig. S7. *Rv*FeMP‐2 and *Rv*FeMP‐1 mutants were purified by the same protocol. For cultivation in a Zn‐rich environment, 1 mm ZnSO_4_ was added when IPTG was added. The sample from the Zn‐rich culture was purified by the same protocol.

### Expression and purification of DRB0118

A codon optimized DNA fragment corresponding to DRB0118 without a signal peptide region (58–337) was purchased from Integrated DNA Technologies (Coralville, Iowa, USA). A DNA fragment of maltose‐binding protein (MBP) was amplified from a pMAL‐c5X vector (New England Biolabs) by using primers shown in Table S2. The DNA fragments of DRB0118 and MBP were inserted into a pET28a vector by the In‐fusion technique, resulting in a construct with an N‐terminal 6xHis tag and a TEV cleavage site (ENLYFQG). The final amino acid sequence is the following, in which italic characters and the underline indicate the MBP and DRB0118 region, respectively.

MGSSHHHHHHENLYFQG*MKIEEGKLVIWINGDKGYNGLAEVGKKFEKDTGIKVTVEHPDKLEEKFPQVAATGDGPDIIFWAHDRFGGYAQSGLLAEITPDKAFQDKLYPFTWDAVRYNGKLIAYPIAVEALSLIYNKDLLPNPPKTWEEIPALDKELKAKGKSALMFNLQEPYFTWPLIAADGGYAFKYENGKYDIKDVGVDNAGAKAGLTFLVDLIKNKHMNADTDYSIAEAAFNKGETAMTINGPWAWSNIDTSKVNYGVTVLPTFKGQPSKPFVGVLSAGINAASPNKELAKEFLENYLLTDEGLEAVNKDKPLGAVALKSYEEELVKDPRIAATMENAQKGEIMPNIPQMSAFWYAVRTAVINAASGRQTVDEALKDAQT* KTNLDATIFNFALNLEYLEAAFYLAAVGRLNELTAAGGDASKVTLPSGVTGMGGTAVPGLTGDLRAMMEEIADDELAHVKVIRSVLGSAAVAQPRLDLSASFLAAGSLASNGAITNFNPYANPLFFLHGAFVFEDVGVTAYKGAARLLVGDKPGGNLENAAGILAVEAYHAGSIRTQLFMRRTEQAAAGLTVEQVVQAISNLRDSVDGADDRDQGITANGNAGVLARDANIIPTDSNGIAFSRTPRQVANIVFLDTTGKAARGGFFPDGLTGDYSSILSL

The expression construct was transformed into *E. coli* BL21(DE3) (New England BioLabs). Transformed cells were grown at 37 °C in LB medium with 50 μg·mL^−1^ kanamycin until OD_600_ became approximately 0.7. The protein expression was induced by the addition of IPTG at a final concentration of 0.5 mm. After 20 h at 18 °C, cells were collected by centrifugation (6000 ***g***, 30 min) at 4 °C and resuspended in a buffer containing 20 mm Tris–HCl pH 8.0 and 300 mm NaCl. Cells were lysed by sonication on ice and cell debris were removed by centrifugation (20 000 ***g***, 60 min) at 4 °C. The supernatant was loaded onto a HiTrap TALON column equilibrated with a buffer containing 20 mm Tris–HCl pH 8.0, 200 mm NaCl, and 5 mm imidazole. The column was then washed by the equilibration buffer. The protein was eluted by a buffer containing 20 mm Tris–HCl pH 8.0, 200 mm NaCl, and 200 mm imidazole. The eluted fraction was mixed with TEV protease and dialyzed against 20 mm Tris–HCl pH 8.0 overnight at 4 °C. The sample was then loaded onto a HisTrap column equilibrated with a buffer containing 20 mm Tris–HCl pH 8.0 and 200 mm NaCl. The flowthrough fraction was collected, concentrated, and loaded onto a Superdex 200 Increase 10/300 SEC column equilibrated with 20 mm Tris–HCl pH 8.0. The fraction containing MBP‐DRB0118 was concentrated and stored at −80 °C. The concentration of the sample was estimated from absorption at 280 nm and the extinction coefficient. High‐resolution clear native PAGE was performed using sample preparation reagent EzApply Native (ATTO, Tokyo, Japan), standard marker EzStandard Native (ATTO), and buffer EzRun ClearNative (ATTO).

### Protein crystallization of *Rv*FeMP‐1

Crystallization was performed by a sitting‐drop vapor diffusion method with MemGold, MemGold 2 (Molecular Dimensions, Maumee, OH, USA) and PEGRx (Hampton Research, Aliso Viejo, CA, USA) kits at a 1 : 1 mixture of *Rv*FeMP‐1 (10 mg·mL^−1^) and well solution at 20 °C. After 2 months, crystals were obtained under the condition A (0.1 m MES pH6.0, 11% w/v PEG 20000). Before a crystal was frozen in liquid nitrogen, it was soaked into the crystallization solution supplemented with 30% (v/v) ethyl glycol (Hampton Research) for cryo‐protection.

We also found other crystals under condition B (0.1 m magnesium chloride hexahydrate, 0.03 m Tris–HCl pH 8.2, and 19% w/v PEG 4000) and condition C (0.4 m sodium thiocyanate 0.1 m sodium acetate pH 4.0 16% w/v PEG4000) after 6 months. The crystal from condition B was soaked into the crystallization solution supplemented with 15% (v/v) trehalose (Molecular Dimensions) before it was frozen in liquid nitrogen. The crystal from condition C was directly frozen in liquid nitrogen. Although the concentration of the precipitant PEG4000 in condition C was not high enough to function as a cryoprotectant, we could freeze the crystal without observation of ice rings.

A sample obtained from the Zn‐rich culture was crystallized under condition B. A crystal was soaked into the crystallization solution supplemented with 20% (v/v) trehalose before it was frozen in liquid nitrogen.

### Data collection and structure determination

Crystals were harvested with LithoLoops (Protein Wave Corporation, Nara, Japan) attached to CrystalCaps (Hampton Research) and stored in Universal V1‐Pucks (MiTeGen, LLC, Ithaca, NY, USA). All X‐ray diffraction experimental data were collected on the BL44XU beamline of SPring‐8, Hyogo, Japan. Diffraction images were obtained at 100K using an EIGER X 16M detector (Dectris, Baden‐Daettwil, Switzerland). The datasets of crystals obtained under condition A were collected at a wavelength of 0.9 Å. The anomalous scattering data were obtained at a wavelength of 1.20 Å. The collected dataset was processed by xds [64] and scaled by aimless [65]. The nominal resolution limit was determined by CC_1/2_ values in the highest resolution shell [66]. Phase determination and model building of *Rv*FeMP‐1 was performed by phenix autosol [67]. The obtained structural model was manually built by coot [68] and structural refinement was performed by refmac5 [69] in the ccp4 suite [70] and phenix.refine in phenix [67]. The datasets of crystals under conditions B and C were processed by xds and phase determination was performed by molecular replacement method using molrep [71] in the ccp4 suite using the solved *Rv*FeMP‐1 structure. Anomalous scattering data was collected from crystals of condition B at wavelengths of 1.30 and 1.28 Å, which are longer and slightly shorter than the K‐edge absorption wavelength of Zn (~ 1.283 Å), respectively. The structure figures in this paper were prepared by pymol (The PyMOL Molecular Graphics System, Version 2.0; Schrödinger, LLC, New York, NY, USA). The stereochemical quality of the final model was checked by molprobity [72]. Data collection and refinement statistics are summarized in Table 1 and Table S1.

### Subcellular localization

The gene of full‐length *Rv*FeMP‐1 was inserted in a pAcGFP1‐N1 vector (Clontech). The expression construct was transfected into human embryonic kidney cells 293 (HEK293, RRID: CVCL_0045) obtained from RIKEN BRC (Tsukuba, Ibaraki, Japan) by lipofectamine method with Lipofectamine 2000 (Thermo Fisher Scientific, Waltham, MA, USA) and incubated in Dulbecco's Modified Eagle Medium with 10% fetal bovine serum (Thermo Fisher Scientific). After 48 h, cells were stained with DAPI (Nacalai Tesque) and Bip/Grp78 (BD Transduction, Franklin Lakes, NJ, USA), visualizing DNA and endoplasmic reticulum respectively. Alexa Fluor546 anti‐mouse antibody was used as a secondary antibody. The cell membrane was stained with tetramethylrhodamine isothiocyanate (TRITC)‐labeled lectin (Sigma‐Aldrich, St. Louis, MO, USA). Fluorescence signal images were captured using a BZ‐X700 system (Keyence, Higashiyodogawa‐ku, Osaka, Japan). All experiments were performed using *Mycoplasma*‐free cells.

### Enzymatic assay

Phosphatase assay using *p*NPP was performed as described below. The mixture contains 10 μg of *Rv*FeMP‐1, 0.1 mm *p*NPP (New England BioLabs), and 20 mm Tris–HCl pH 8.0 in a total volume of 50 μL. The solution was mixed at 37 °C and 300 r.p.m. by ThermoMixer C (Eppendorf, Hamburg, Germany). The reaction was then quenched by the addition of 1 m NaOH. Absorbance at 405 nm was measured in a 1 mL quartz cuvette (*l* = 1 cm) using Spectramax M2 (Molecular Devices, San Jose, CA, USA).

General substrate screening with various phosphorylated metabolites were performed using BIOMOL® Green Reagent (BioAssay Systems, Hayward, CA, USA), which is used to quantify free phosphoric acid released from phosphorylated compounds. The mixture contains 10 μg of *Rv*FeMP‐1, 0.1 mm substrate, and 20 mm Tris–HCl pH 8.0 in a total volume of 50 μL. For *Rv*FeMP‐2 and DRB0118, the mixture contains the sample of 4 and 2 μg, respectively. The solution was mixed at 37 °C and 800 r.p.m. After 2 h, the reaction was terminated by the addition of 100 μL of malachite green reagent and the solutions were incubated for 30 min at room temperature. The presence of free phosphoric acid makes the solution green. Then, the amount of inorganic phosphate was measured by using absorbance at 620 nm in a 96‐well microplate using Spectramax M2. The amount of released phosphoric acid was quantified by a standard carve and the activity (nmol·min^−1^·mg^−1^ protein) was calculated. As an exception, for the assay of DRB0118 using G6P, GTP, and *p*NPP, the mixture contains 2 μg of DRB0118, 1 mm substrate, 20 mm Tris–HCl pH 8.0 in a total volume of 50 μL. The solution was mixed at 37 °C and 800 r.p.m. for 1 h. Other than this substrate (G6P, GTP, and *p*NPP) and protein (DRB0118) combination, the reaction conditions are the same as for *Rv*FeMP‐1 above. These assays were replicated three times. The phosphatase activity of *Rv*FeMP‐1 mutant was also investigated according to the same protocol. 8‐oxo‐dGTP, N6‐methyl‐dATP, and 2‐OH‐dATP were purchased from Jena Bioscience, Jena, Germany. Abbreviations of compounds were shown below. Eryth‐4P, erythrose‐4‐phosphate; F16P, fructose‐1,6‐bisphosphate; F6P, fructose‐6‐phosphate; G1P, glucose‐1‐phosphate; G6P, glucose‐6‐phosphate; Gal‐1P, galactose‐1‐phosphate; IP3, inositol trisphosphate; Man‐6P, mannose‐6‐phosphate; P‐Cho, phosphocholine; Rib‐5P, ribose‐5‐phosphate.

## Conflict of interest

The authors declare no conflict of interest.

## Author contributions

SK and YFukuda designed research; SK, KD, YFukuda, and MO performed experiments; SK, YFukuda, MO, YFujio and TI analyzed data; and SK, YFukuda, and MO wrote the paper.

### Peer review

The peer review history for this article is available at https://www.webofscience.com/api/gateway/wos/peer‐review/10.1111/febs.17296.

## Supporting information


**Fig. S1.** Fluorescence signal images of GFP‐fusion *Rv*FeMP‐1 expressed in HEK293T cells.
**Fig. S2.** The detailed description of the interaction in linker regions.
**Fig. S3.** High resolution clear native PAGE gel of DRB0118.
**Fig. S4.** Anomalous scattering data of *Rv*FeMP‐1 cultured in Zn‐rich medium.
**Fig. S5.** Superimposition of apo and Zn‐Mg *Rv*FeMP‐1 structure.
**Fig. S6.** Biochemical significance of this enzymatic reaction.
**Fig. S7.** The result of size exclusion chromatography and SDS‐PAGE of *Rv*FeMP‐1.
**Table S1.** Data collection of anomalous data.
**Table S2.** Primers used in this research (from 5′ to 3′).

**Data S1.** Sequences used for alignment (supporting Fig. 1B).

**Data S2.** Sequences used for alignment (supporting Fig. 1C).

## Data Availability

The raw diffraction data collected in this study are available at the Xtal Raw Data Archive (https://xrda.pdbj.org) under the IDs corresponding to the Protein Data Bank depositions. The coordinate files and the structure factor files are deposited in the Protein Data Bank (PDB IDs: 8KCE for the Zn/Mg structure, 8W9K for the apo structure, and 8WAI for the Zn/Zn structure).
