# Supplementary material for: Metabolite phosphatase from anhydrobiotic tardigrades
Source: FEBS J. 2024 Oct 17;291(23):5195–213. doi: 10.1111/febs.17296 (PMC11616004; doi:10.1111/febs.17296)
Supplement: Supplementary file 1 — Fig. S1. Fluorescence signal images of GFP‐fusion RvFeMP‐1 expressed in HEK293T cells. Fig. S2. The detailed description of the interaction in linker regions. Fig. S3. High resolution clear native PAGE gel of DRB0118. Fig. S4. Anomalous scattering data of RvFeMP‐1 cultured in Zn‐rich medium. Fig. S5. Superimposition of apo and Zn‐Mg RvFeMP‐1 structure. Fig. S6. Biochemical significance of this enzymatic reaction. Fig. S7. The result of size exclusion chromatography and SDS‐PAGE of RvFeMP‐1. Table S1. Data collection of anomalous data. Table S2. Primers used in this research (from 5′ to 3′). [file FEBS-291-5195-s002.pdf]

Supplementary Information for

Metabolite phosphatase from anhydrobiotic tardigrades

Subaru Kato, Koki Deguchi, Masanori Obana, Yasushi Fujio, Yohta Fukuda, Tsuyoshi Inoue

E-mail: t_inoue@phs.osaka-u.ac.jp

This file includes the following contents:

1. Supplementary Tables S1 to S2

2. Supplementary Figures S1 to S7

1. Supplementary Tables

Table S1. Data collection of anomalous data.

	*Rv*FeMP-1 Zn/Mg	*Rv*FeMP-1 Zn/Mg	*Rv*FeMP-1 Zn/Zn	*Rv*FeMP-1 Zn/Zn (Zn-medium)
Crystallization condition	B		A	
**Data collection**				
Diffraction source	SPring-8 BL44XU			
Wavelength (Å)	1.28	1.30	1.20	1.28
Space group	*C*2		$P2_12_12_1$	
a, *b*, *c* (Å)	89.37 63.96 45.98	89.38 63.91 45.96	60.97 82.22 145.22	61.20 82.81 146.60
α, β, γ (°)	90.00 109.57 90.00	90.00 109.53 90.00	90.00 90.00 90.00	90.00 90.00 90.00
Resolution (Å)	43.32-1.50 (1.53-1.50)	43.32-1.50 (1.53-1.50)	48.98-2.40 (2.49-2.40)	46.98-3.30 (3.56-3.30)
Total reflections	259543	258076	378829	71230
Unique reflections	38983 (1962)	38984 (1938)	29358 (3047)	11767 (2364)
Completeness (%)	99.7 (99.5)	99.8 (98.7)	100.0 (100.0)	99.9 (99.9)
Redundancy	6.7 (6.6)	6.6 (6.2)	12.9 (12.5)	6.1 (6.4)
$I/\sigma(I)$	30.1 (5.8)	22.2 (3.3)	21.0 (8.5)	10.9 (7.0)
$CC_{1/2}$	1.000 (0.960)	1.000 (0.859)	0.998 (0.983)	0.993 (0.978)
R_{merge} (all I+ & I-)	0.037 (0.282)	0.042 (0.540)	0.106 (0.342)	0.124 (0.224)
R_{meas} (all I+ & I-)	0.040 (0.307)	0.046 (0.591)	0.110 (0.356)	0.136 (0.245)
R_{pim} (all I+ & I-)	0.015 (0.119)	0.018 (0.234)	0.031 (0.100)	0.056 (0.098)

Statistics for the highest-resolution shell are shown in parentheses.

Table S2. Primers used in this research (from 5’ to 3’).

Primers to amplify *Rv*FeMP-1 fragment	
Forward	GGAGGCAACAGGGGCATGT
Reverse	TTAAACGCCGGGAACGGC
Primers to amplify *Rv*FeMP-2 fragment	
Forward	GTCAGCGATGTGGACGTCCTT
Reverse	TCAGACAGCATCGCCACG
Primers to linearize pET28a for RvFeMP-1	
Forward	GTTCCCGGCGTTTAAAGCAGCGGCCT GGTGC
Reverse	GCCCCTGTTGCCTCCTCCCTGAAAAT ACAGGTTTTCGTGATGATGATGATGAT GGCTGCT
Primers to linearize pET28a for RvFeMP-2	
Forward	GGCGATGCTGTCTGAAGCAGCGGCCT GGTGC
Reverse	GTCCACATCGCTGACTCCCTGAAAAT ACAGGTTTTCGTGATGATGATGATGAT GGCTGCT
Primers to make *Rv*FeMP-1 D135A	
Forward	GCAAAACCGGCCTACCTTGG
Reverse	GGCGTTCTCCAGAGTGCGG
Primers to make *Rv*FeMP-1 D135N	
Forward	AATAAACCGGCCTACCTTGGCG
Reverse	GGCGTTCTCCAGAGTGCGG
Primers to amplify an MBP fragment for the DRB0118 construct	
Forward	CATCATCATCATCACGAAAACCTGTAT TTTCAGGGCATGAAAATCGAAGAAGG TAAACTGGTAATCTGG
Reverse	GTCAAGGTTGGTCTTAGTCTGCGCGT CTTTCAGGGC
Primers to amplify a DRB0118 fragment	
Forward	AAGACCAACCTTGACGCGACCA
Reverse	CATGCTAGCCATATGTTACAGGCTGAG
Primers to linearize pET28a for DRB0118	
Forward	CATATGGCTAGCATGACTGGTGGAC
Reverse	GTGATGATGATGATGATGGCTGCT

2. Supplementary Figures

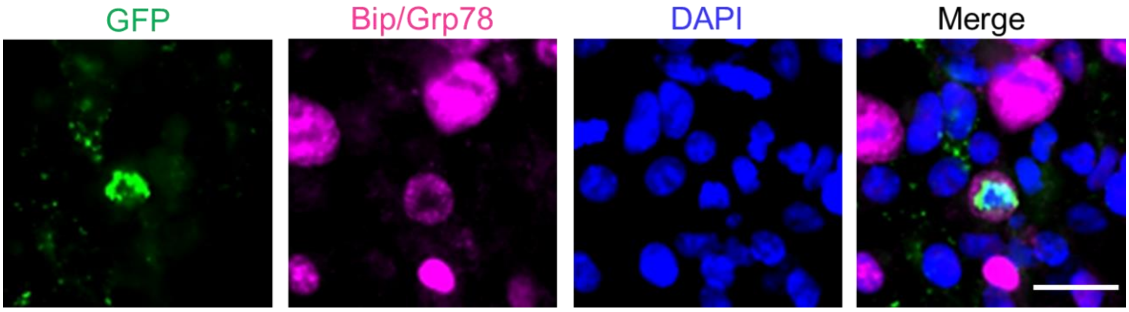

Fig. S1. Fluorescence signal images of GFP-fusion *Rv*FeMP-1 expressed in HEK293T cells. Each cell was co-stained by Bip/Grp78 (ER) and DAPI (nucleus). The scale bar indicates 25 µm.

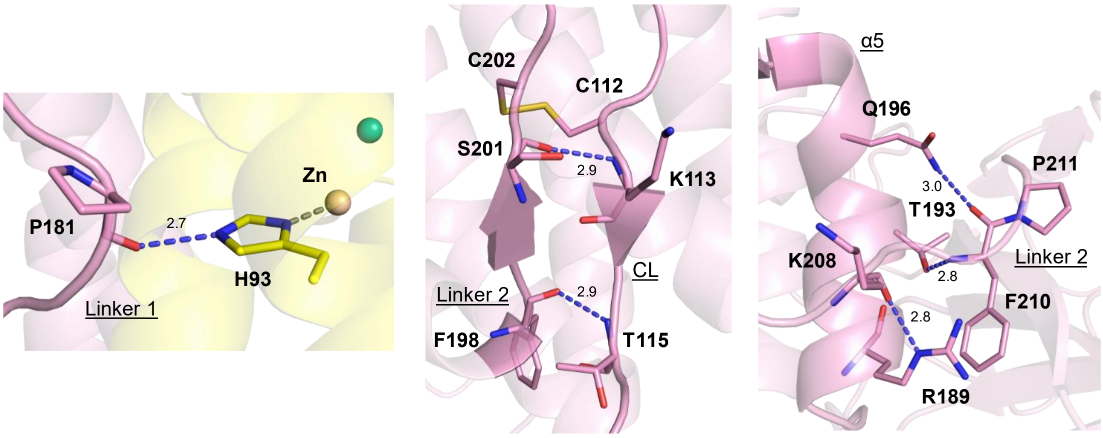

Fig. S2. The detailed description of the interaction in linker regions.

The figures show the interactions between Linker 1 and His93 (left), Linkers 2 and CL (middle), and Linker 2 and α5 (right). Coordination and hydrogen bonds are shown by gray and blue dashed lines, respectively. Numbers in the figure indicate the distance (Å) of hydrogen bonds.

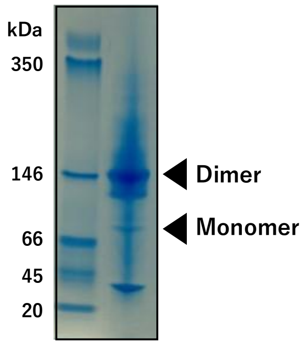

Fig. S3. High resolution clear native PAGE gel of DRB0118.

The molecular weight of MBP-DRB0118 monomer is 69.3 kDa.

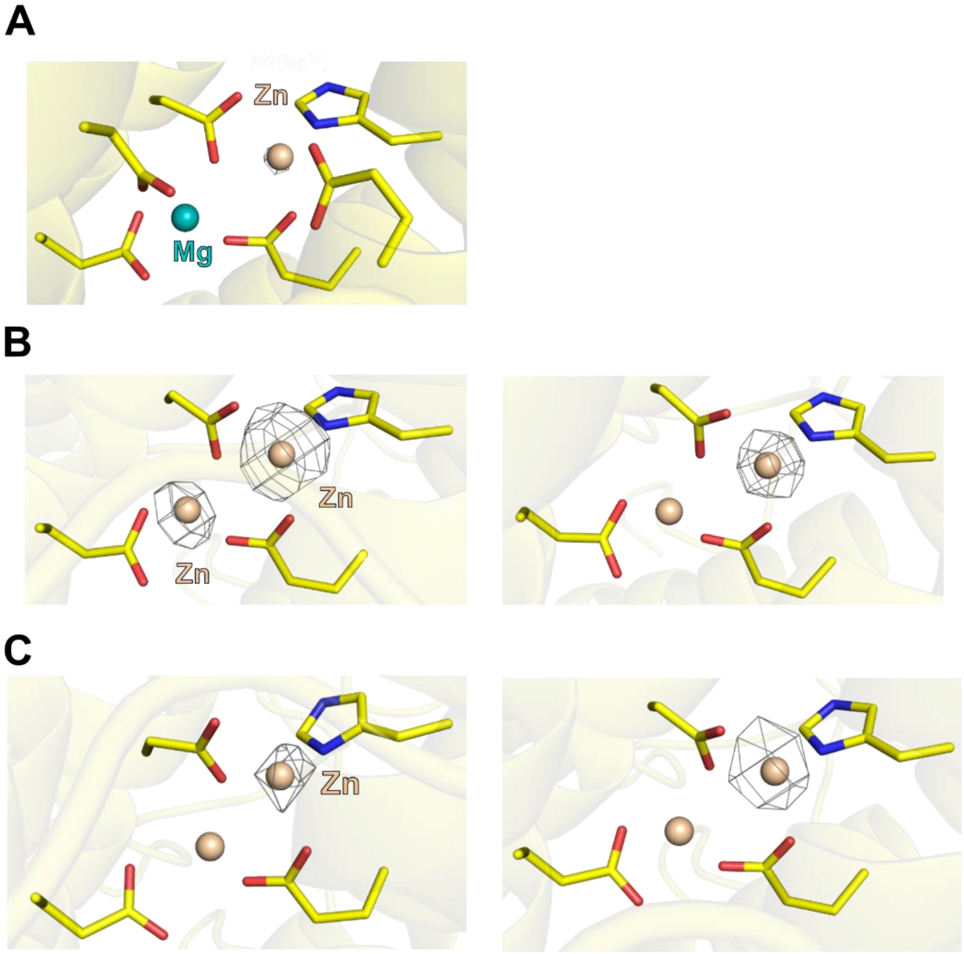

Fig. S4. Anomalous scattering data of *Rv*FeMP-1 cultured in Zn-rich medium.

(A) Anomalous scattering signals from the Zn-Mg *Rv*FeMP-1 data. Anomalous peaks are shown as gray meshes contoured at 10 σ. The wavelength used for X-ray data collection is 1.30 Å. (B) Anomalous scattering signals from the Zn-Zn *Rv*FeMP-1 data. Anomalous peaks are shown as gray meshes contoured at 7 σ (left) and 15 σ (right). The wavelength used for X-ray data collection is 1.20 Å. (C) Anomalous scattering data of *Rv*FeMP-1 cultured in Zn-rich medium. Anomalous peaks observed in chain A (left) and chain B (right) of the asymmetric unit are shown as gray meshes contoured at 4 σ. The wavelength used for X-ray data collection is 1.28 Å.

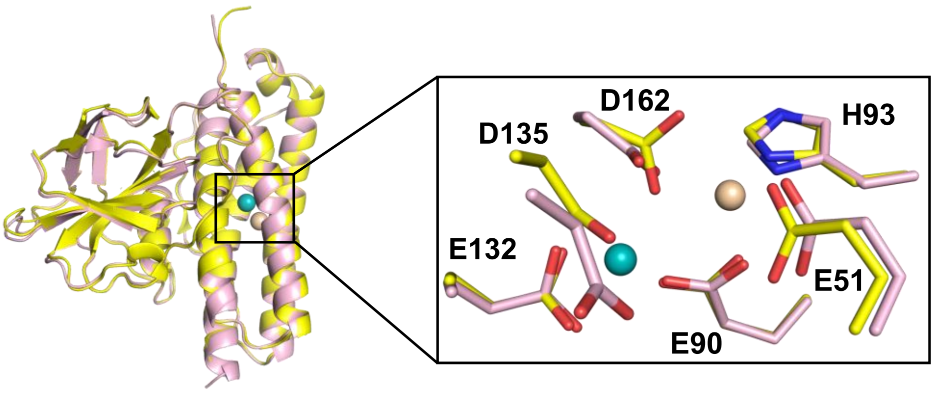

Fig. S5. Superimposition of apo and Zn-Mg *Rv*FeMP-1 structure.

Apo *Rv*FeMP-1 and Zn-Mg *Rv*FeMP-1 are shown by light pink and yellow, respectively.

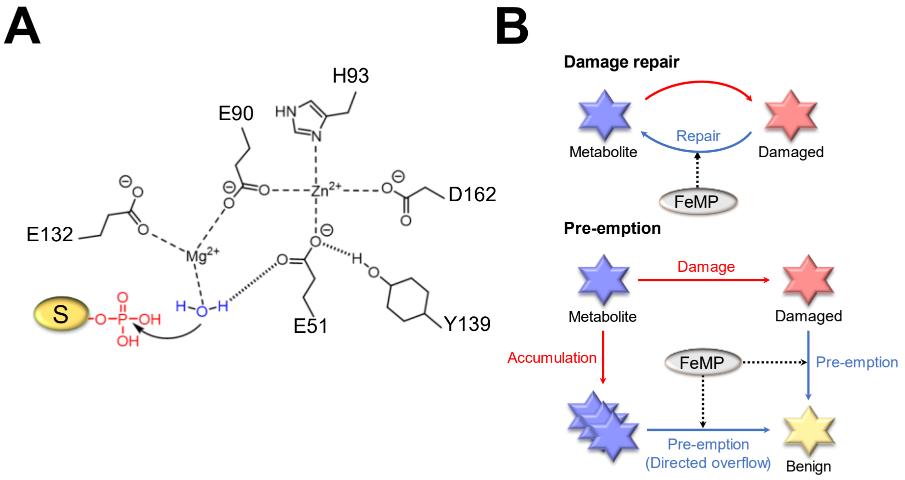

Fig. S6. Biochemical significance of this enzymatic reaction.

(A) Active site of *Rv*FeMP-1 and proposed reaction mechanism. A phosphate group of a substrate is shown by red. The water molecule involved in dephosphorylation is shown by blue. Dashed lines indicate coordination bonds. Doted lines show hydrogen bonds. (B) Schematic view of the possible functions of Tar-fers in the control of damaged metabolites.

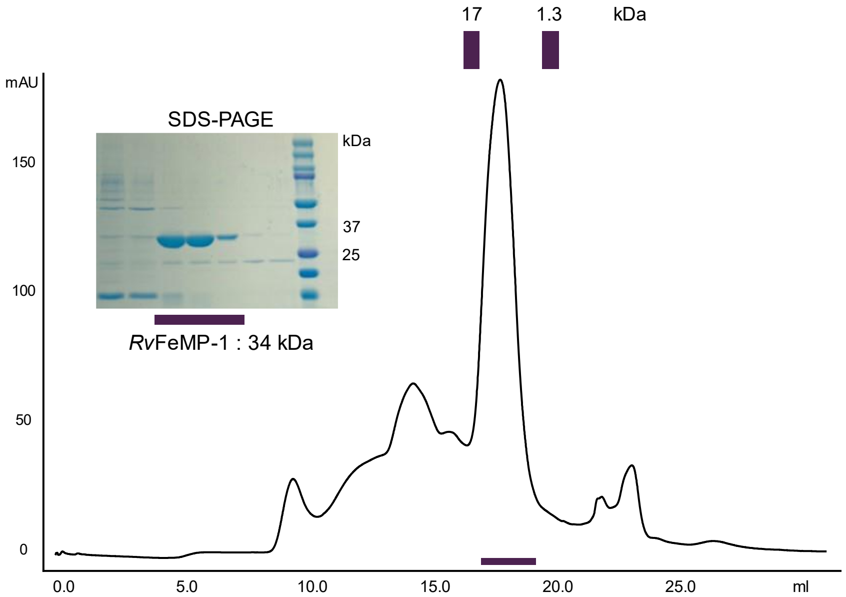

Fig. S7. The result of size exclusion chromatography and SDS-PAGE of *Rv*FeMP-1.
