# Supplementary material for: Metabolite phosphatase from anhydrobiotic tardigrades
Source: FEBS J. 2024 Oct 17;291(23):5195–213. doi: 10.1111/febs.17296 (PMC11616004; doi:10.1111/febs.17296)
Supplement: Supplementary file 2 — Data S1. Sequences used for alignment (supporting Fig. 1B). [file FEBS-291-5195-s001.pdf]

Ferritin-like domains used for phylogenetic analysis in Fig. 1B are written in blue letters.

>GAU94437.1 hypothetical protein RvY_06210 [Ramazzottius varieornatus] up regulated; ferritin-like; signal peptide; IgG like
MNCIKVALFVAACLVGGGLGGGNRGMSRNQDAYAEKMDMTLDALNFLLGVEHLASAFYV
QAVNNFTADDFKAAGLAQRDYDQFVGVRNNEVDHRDTLISVIKSLGGKPNPPCKYTFPVT
DVASVLKVSRTLENADKPAYLGALRDIKSVELRTSVQGALSGDSAHAAFFAYLTGKAPAPG
PVDGPLTQRHIATLAQDFIVSCPYPAPKPFPKLTLSPQSGPVGTVVATTCAQDVDTNGVMC
AIISGNQGTLMQRPGQAKDGSGAATCTIPPGVKGILFIAWVRGRDVLNVGVDDSSTVCGP
NYFLLSALGDAVPGV

>tr|A0A1D1W2V1|A0A1D1W2V1_RAMVA Uncharacterized protein OS=Ramazzottius varieornatus OX=947166 GN=RvY_17634-1 PE=4 SV=1 RvY_17634
MLELILLFLAGSYAAPVHVSDVDVLQFALTVENLASTFYIQGLQKHPKEEFLNAGVKEADYD
QIVRVRDNEAGHRDTLKAAIQKLGGTPNPPCQYKFPDDDIPSFLKVARTLENADIPAYTGSI
KDLTDKRLITAAGTIVTVDARHAAFFNHITGKAPAPASFDIPLGQRQISSLAKQFIVSCPHPIP
EPFPELKLTPESGPAGSAVTLATSAPLRGVNCAIITATGITFSPVQDNKCTIPQASGGVYVVL
TSASDSRGLSDDNTLGIAPFIVAARGDAV

>OWA52935.1 hypothetical protein BV898_17377 [Hypsibius exemplaris]
MVGITFIVGYSCLLCSVLGAPYGYLYGSSGTAPVTAAPGGGGGGGGGGGATTPGKPVYNS
TADISVLQYALTLENLEAAFYVDAVGKHTSAEFQAIGLTDRDYQILVNVRDHEVTHVAALSAA
IAGLGATPVPACTYKFPATDVKTLLAIARALEKTGVSAYDAAAQDISNPAYLTVAATIVTVEAR
HAAFFNYITGKNPASAPFDIPLGRRQIVTLASPFIASCPYDLPAPFAGLTISPASGPAGISLALT
TSPAGLVSTAGVQCAFITGSGKNWLVPVVGGACAVPATLTGEVYVVLTSAASIDALNDSNTL
AGPASFAVPDKGDVAA

>sp|P22242|DRPE_CRAPL Desiccation-related protein PCC13-62 OS=Craterostigma plantagineum
MAQQPTFASAALVSFFLALICSCSYAAWHHEKDDIPKSDVSLLEFPLNLELLEAEFFAWAAF
GKGIDELEPELAKGGPSPIGVQKANLSPFIRDIIAQFAYQEFGHVRAIQSSVEGFPRPLLDLS
AKSFATVMDSAFGKTLKPPFDPYANDINYLLACYVVPYVGLTGYVGANPKLESPVSRKLVA
GLLAVEAGQDAIIRALLYERATDKVEPYGITVAEFTNKISELRNKLGDKGVKDLGLIVEPELG
AEGKISGNVLAGDKNSLAFPRTPERCLGSCTAAAMRPSPAAFIPKAPTGKSPSLIWRIRAFS
IV

>tr|A0A2U8JD39|A0A2U8JD39_9LAMI 13-62 protein OS=Lindernia brevidens OX=263965 PE=4 SV=1

MAQQRPTIAAALTISFCLLMQLCSCSLFSHDLPKSDVNMLEFPLNLEYLEAEFFSWAALGK
GLDELEPDLAKGGPPPIGAKKAKLSDVVRDIVEQFAYQEFGHVRAIQSSVPGFPRPLLDLS
EKSFATIMDDAFGKPLNPPFDPYANDINYLLAAYVVPYVGLTGYVGANPKLESPKSRKLVAG
LLGVESGQDAVIRALLYERKMEKVEPYDITVAEFTNKISELRNKLGSKGVKDKGLLVEPELG
AEGKTSGNILAGDKNSLSFERTPEEVLRIVYGSGDEGKPGGFYPKGADGHIAKSHLEHESF
ISMVL

>tr|A0A010Q9S5|A0A010Q9S5_9PEZI Rds1 OS=Colletotrichum fioriniae PJ7

MAPRSMFRLLALASGASAVPFVSEPQTTVTSEPTITPSQVAVTNVTSHGPYTGPSPTTTGA
LSTSVLASEVPILPPPDDAYDYPADGKLHGDQPAPYTPSGGIGTNGSAPVYRVQSDFDYQ
SLALALYQEYIELDLFHWGLATFSDEEFEELGLNAEDRYLLQFMAEQEIGHATVITNMLGAQ
APKQCTYNYPVTNLREYIDFNQKLTRWGESGVYGFLPHLNSGPAAQLLLQSITTEARQQMI
FRQFEGLFPMPEWHIPGIPQSWAWTLLAPYISSCPADQTRLIWQNFPALHILNQPNPYRIN
GSNVWNETTGGWANTAATTNITDAESCVNATDPLQDCNAAITQNRTMPLSYPGRQVFFQ
WDAPGQAVGPNNSYITATNVVEPKFAAWVSQLNVTFSPLMNVSLESRTAYTIQPNVSTWE
HDPAINSTMFVALTDTDMYVTAHNLTMINPHVAALAVYQAG

>sp|P53693|RDS1_SCHPO Protein rds1 OS=Schizosaccharomyces pombe (strain 972 / ATCC 24843)

MVQALTASLMAGALLARGIIGAKADPVNFAGIGGAAYEYNYTATGSFNQSIMPANFTPAGGI
DTNDSSPTYHPFSDFDYQSLSLALYHEYIEYDLFNYGLTKFSDAEFDEAGIDAEYRHLIRFM
AQQEIGHIELVTNMLGPNAPKACSYQYNFDTVGSFIDFAQTLTKWSESGVYGFLPHLDSRA
AAALLLQSITTEARQQMSLRQLQGLFPYPVWFETGIPQSFAWSLIAPFIVGCPAENEKLVW
QNFPALHLVSPPVHTNFTNGSFPQYPNGTYMYPAAVSTNRTFPLSLPGQSVELAWDAPGM
AVGPNSSYITSTSAGTPRYAAWISQLNVTYAPLNITGNNSGVTYQPSSHLYNDSTQQVINGT
NFLVLVDEAIPVTPFNITAINEHVVAGPLVYESG

>tr|Q9RZK8|Q9RZK8_DEIRA Dessication-associated protein OS=Deinococcus radiodurans DR_B0118

MSTPVEPLLCLSSVCDTRKAMKEEMQSTRRRFLGMAGAMGAGAVLAGCANVGASEPTKT
NLDATIFNFALNLEYLEAAFYLAAVGRLNELTAAGGDASKVTLPSGVTGMGGTAVPGLTGDL
RAMMEEIADDELAHVKVIRSVLGSAAVAQPRLDLSASFLAAGSLASNGAITNFNPYANPLFF
LHGAFVFEDVGVTAYKGAARLLVGDKPGGNLENAAGILAVEAYHAGSIRTQLFMRRTEQAA
AGLTVEQVVQAISNLRDSVDGADDRDQGITANGNAGVLARDANIIPTDSNGIAFSRTPRQV

ANIVFLDTTGKAARGGFFPDGLTGDYSSILSL

>1R03 human mitochondrial ferritin
PAAGPSRVRQNFHPDSEAAINRQINLELYASYVYLSMAYYFSRDDVALNNFSRYFLHQSRE
ETEHAEKLMRLQNQRGGRIRLQDIKKPEQDDWESGLHAMECALLLEKNVNQSLLELHALA
SDKGDPHLCDFLETYYLNEQVKSIKELGDHVHNLVKMGAPDAGLAEYLFDTHTLGNENKQ
N

>1BFR bacterioferritin E.coli
MKGDTKVINYLNKLLGNELVAINQYFLHARMFKNWGLKRLNDVEYHESIDEMKHADRYIERI
LFLEGLPNLQDLGKLNIGEDVEEMLRSDLALELDGAKNLREAIGYADSVHDYVSRDMMIEIL
RDEEGHIDWLETELDLIQKMGLQNYLQAQIREEG

>DPS Listeria innocua serovar 6a
MKTINSVDTKEFLNHQVANLNVFTVKIHQIHWYMRGHNFFTLHEKMDDLYSEFGEQMDEV
AERLLAIGGSPFSTLKEFLENASVEEAPYTKPKTMDQLMEDLVGTLELLRDEYKQGIELTDK
EGDDVTNDMLIAFKASIDKHIWMFKAFLGKAPLE

>HUMAN Ribonucleoside-diphosphate reductase Homo sapiens
MLSLRVPLAPITDPQQLQLSPLKGLSLVDKENTPPALSGTRVLASKTARRIFQEPTEPKTKA
AAPGVEDEPLLRENPRRFVIFPIEYHDIWQMYKKAEASFWTAEEVDLSKDIQHWESLKPEE
RYFISHVLAFFAASDGIVNENLVERFSQEVQITEARCFYGFQIAMENIHSEMYSLLIDTYIKDP
KEREFLFNAIETMPCVKKKADWALRWIGDKEATYGERVVAFAAVEGIF

>1JKU Manganese Catalase from Lactobacillus plantarum
MFKHTRKLQYNAKPDRSDPIMARRLQESLGGQWGETTGMMSYLSQGWASTGAEKYKDL
LLDTGTEEMAHVEMISTMIGYLLEDAPFGPEDLKRDPSLATTMAGMDPEHSLVHGLNASLN
NPNGAAWNAGYVTSSGNLVADMRFNVVRESEARLQVSRLYSMTEDEGVRDMLKFLLARE
TQHQLQFMKAQEELEEKY
