# Supplementary material for: Metabolite phosphatase from anhydrobiotic tardigrades
Source: FEBS J. 2024 Oct 17;291(23):5195–213. doi: 10.1111/febs.17296 (PMC11616004; doi:10.1111/febs.17296)
Supplement: Supplementary file 3 — Data S2. Sequences used for alignment (supporting Fig. 1C). [file FEBS-291-5195-s003.pdf]

>GAU94437.1 hypothetical protein RvY_06210 [Ramazzottius varieornatus]
MNCIKVALFVAACLVGGGLGGGNRGMSRNQDAYAEKMDMTLDALNFLLGVEHLASAFYV
QAVNNFTADDFKAAGLAQRDYDQFVGVRNNEVDHRDTLISVIKSLGGKPNPPCKYTFPVT
DVASVLKVSRTLENADKPAYLGALRDIKSVELRTSVQGALSGDSAHAAFFAYLTGKAPAPG
PVDGPLTQRHIATLAQDFIVSCPYPAPKPFPKLTLSPQSGPVGTVVATTCAQDVDTNGVMC
AIISGNQGTLMQRPGQAKDGSGAATCTIPPGVKGILFIAWVRGRDVLNVGVDDSSTVCGP
NYFLLSALGDAVPGV

>tr|A0A1D1W2V1|A0A1D1W2V1_RAMVA Uncharacterized protein OS=Ramazzottius varieornatus RvY_17634
MLELILLFLAGSYAAPVHVSDVDVLQFALTVENLASTFYIQGLQKHPKEEFLNAGVKEADYD
QIVRVRDNEAGHRDTLKAAIQKLGGTPNPPCQYKFPDDDIPSFLKVARTLENADIPAYTGSI
KDLTDKRLITAAGTIVTVDARHAAFFNHITGKAPAPASFDIPLGQRQISSLAKQFIVSCPHPIP
EPFPELKLTPESGPAGSAVTLATSAPLRGVNCAIITATGITFSPVQDNKCTIPQASGGVYVVL
TSASDSRGLSDDNTLGIAPFIVAARGDAV

>OWA52935.1 hypothetical protein BV898_17377 [Hypsibius exemplaris]
MVGITFIVGYSCLLCSVLGAPYGYLYGSSGTAPVTAAPGGGGGGGGGGGATTPGKPVYNS
TADISVLQYALTLENLEAAFYVDAVGKHTSAEFQAIGLTDRDYQILVNVRDHEVTHVAALSAA
IAGLGATPVPACTYKFPATDVKTLLAIARALEKTGVSAYDAAAQDISNPAYLTVAATIVTVEAR
HAAFFNYITGKNPASAPFDIPLGRRQIVTLASPFIASCPYDLPAPFAGLTISPASGPAGISLALT
TSPAGLVSTAGVQCAFITGSGKNWLVPVVGGACAVPATLTGEVYVVLTSAASIDALNDSNTL
AGPASFAVPDKGDVAA

>CAF1188151.1 unnamed protein product [Adineta steineri]
MDAAPYAHITQGPAIVQPTGPAVITAPRRGINPLIPIIGIGLVLFLIAATIILSLIPTYLSTRNIDPV
DSYRYNTVYQINNGFLPIGALSADNCATIQTDQQALSGSIRGTSSPTVTVISCAIVGNNTDS
GRRRRAVLQPKGNFISINGRATGNGICLRGACLQQRVARFIELLLNRGSFVLTLVGAQYSLK
VISATILPTETPDLTNDLAILNYALVLEQLEANFYTQFQAQFTAQNFIAAGFTQTTYDYFNIIYF
HELAHVRALTAVISQLGGTPVSQCTYNFAAVTDVKSYVSVAQALENTGAMAYDGAVNGIAN
PMIRKTAATIATVEARHAAYLNTLNGASPFPNTVENATVPALVIAAIQKFLISCPFTITPPTVPYI
GSSSFTGTVPTSNDTKVSPYTSAMYTNDMKVLNYALVAENLEAAYYNKYVSTYSSTDYTN
NGFPDASIYFILIREHENAHVQILQTVIKQRGGSPVSVCTYTFPVTDIRSFISLSRTFENTGVS
AYTGAIDKIRDPSIILAAATIATVEARHASYLNSLSGGVPFPDVTDTPIEPTQIAATLAQFQTCP
FPSDLILPV

>sp|P22242|DRPE_CRAPL Desiccation-related protein PCC13-62 OS=Craterostigma plantagineum
MAQQPTFASAALVSFFLALICSCSYAAWHHEKDDIPKSDVSLLEFPLNLELLEAEFFAWAAF
GKGIDELEPELAKGGPSPIGVQKANLSPFIRDIIAQFAYQEFGHVRAIQSSVEGFPRPLLDLS
AKSFATVMDSAFGKTLKPPFDPYANDINYLLACYVVPYVGLTGYVGANPKLESPVSRKLVA
GLLAVEAGQDAIIRALLYERATDKVEPYGITVAEFTNKISELRNKLGDKGVKDLGLIVEPELG
AEGKISGNVLAGDKNSLAFPRTPERCLGSCTAAAMRPSPAAFIPKAPTGKSPSLIWRIRAFS
IV

>tr|A0A2U8JD39|A0A2U8JD39_9LAMI 13-62 protein OS=Lindernia brevidens
MAQQRPTIAAALTISFCLLMQLCSCSLFSHDLPKSDVNMLEFPLNLEYLEAEFFSWAALGK
GLDELEPDLAKGGPPPIGAKKAKLSDVVRDIVEQFAYQEFGHVRAIQSSVPGFPRPLLDLS
EKSFATIMDDAFGKPLNPPFDPYANDINYLLAAYVVPYVGLTGYVGANPKLESPKSRKLVAG
LLGVESGQDAVIRALLYERKMEKVEPYDITVAEFTNKISELRNKLGSKGVKDKGLLVEPELG
AEGKTSGNILAGDKNSLSFERTPEEVLRIVYGSGDEGKPGGFYPKGADGHIAKSHLEHESF
ISMVL

>tr|Q10LK1|Q10LK1_ORYSJ Desiccation-related protein PCC13-62, putative, expressed OS=Oryza sativa subsp. japonica
MNNLQSEMASPRICCSALLVLLLVSSCNAGDHPACPAAWSAAVGAELFHGGVGGGGGE
AQCSAAAPHTPVAVFAHDVDPVRFALNLEFAEAEFFLHAAFGVGLDHLAPNLTLGGPPPVG
ARKAGLDELTWRVCAEFAYQEIGHLRAIQRTVGGIPRPLIDLSAHNFARVMDEAVGYHLDP
PFDPDANSLNFLLAVYVIPYLGINGYTGTNPLIDGYATKRLVAGLLAVESGQDAVVRGLLFEH
RRETVSPYGATVAELTDRVSALRNKLGQCGVKDEGLIVPEQLGAEGKICTNILSANVDSLSY
SRTPAELLRILYLTGDEHVPGGFYPEGANGRIARMFLKKPPRINHGV

>tr|A0A2G2YIX3|A0A2G2YIX3_CAPAN Desiccation-related protein PCC13-62 OS=Capsicum annuum
MILVYFILLFFLQLSSNSVANENFENYVGCDMPKSDVDLVEFPLNLEYLEAEFFLWGSLGYG
LDKFAPELADSGPPPIGAQIAKLSPLIKDVITQFGFQEVGHLRAIKDTITGFPRLLLNLSRESF
ATVMDDAIGHPLKPPFDAYANDINYLLASYVIPYVGLTGYVGANPKLQSSTAKRLVAGLLGV
ESGQDAVLRALLYERGREKVEPYGITVAEFTNRISKLRNKLGRHGIKDEGLNVKPKVGAEG
RIRGNVLAGDKYSMAYDRTPKEILRIVYGSGKENKPGGFYPTGAEGAIAKSYLRHGD

>tr|A0A199UZX2|A0A199UZX2_ANACO Desiccation-related protein PCC13-62

OS=Ananas comosus
MGVLNYSVSYASLFLFFVFATIPSPLFAQIFKELSHHHHHHRHRGGVHVALPESDVELLEFP
LNLEYLEAEFFLWGALGHGLDVVAPNLTEGGPSPVGARKAALDPLILDVVTQFAYQEVGHI
RAIKKRVKGFPRPLLDLSATNFAKTVNNALNRELNPPFDPYANGLNFLLASYLIPYVGLTGY
VGANPKLKSARAKRLVAGLLAVESAQDTVIRTLLYERLLTKVYPYDFTVAEFTNRASHLRNK
LGKSGVKDEGLVVPPVLGAEGKIQGNIIAGDRYSTAYDRTPEEILSIVYASGNASVPGGFYP
NGARGQIAESYIKRA

>tr|A0A022QNZ3|A0A022QNZ3_ERYGU Desiccation-related protein PCC13-62
OS=Erythranthe guttata (Yellow monkey flower)
MKMVHVFSASNVFKFVFIILASIEQTAVVKSNPLCPSEYPKFGVPIYKGDIDMLQFAENLEHL
EADFFLWSSLGYGLDKIAPELVMGGPPPIGAQKANLDFLTENIIREFAFQEVGHLRALKTTV
GGFPRPLLNLSAENFANIFNEAFGYELVPPFDPYRNSLSYMLASYVVPYVGLVGYVGTNPK
LIGYVSKRLLVGLMGVEAGQDAVIREYLYERAAKVVHPYNHTVAEFTIRISELRNRLARCGIK
DEGIIVPPFLGAENRTVSNVLSADYNSLSYSRTPPEILRIVYGTGSECVPGGFFPKGGNGRI
ARQFLDKPSN

>KAB8760602.1 hypothetical protein FH972_026594 [Carpinus fangiana]
MKFTAVTAALVGSALATPFSFPLSNGFPNPSTDVIGLIQQHAGGTLPNTPLPKDVTPDVVTA
LQLIAANEIFEVAYFTELYHNITNHIHGYDLAKYNKTFVVDAIHRIRAQEELHGLGANAILKSV
GSQPIPPCKYEFPVTTFEDAIALANTFTDVVLGVLPQVQLLGGTKGGKDGSSLIPLIGSILGQ
EAEQDGWFRTLLGKPPSAAPFLTPTTPQFALAAVSMFLVPGSCPPVYNDITKKIGALAPLTV
ASISEQKSEAQFSVKGKLDAKHNALVYVSGQSAPVTVDIQNVQTKGGVTTFSAAFPYKITA
TSGFAEGLSVAAVTKCKRAYANVADVATDTLFGPGLIERH

>KAH9815944.1 ferritin-like domain-containing protein [Melampsora americana]
MISNSLKASVFLVACVVSASFEKRDKVLTDVDILNFALTLEHLDSAFYSQGLQKYDQDAFTN
AGFSMSTRQSIQSISNDEAAHVSFLTSALQAAGAIPVQACNYTFPYTDVKSFLAVSQILEGV
GVSAFLGAAASIKNLNYLTAAGSILTDEARHNAYLRFVNGESPFPTAFDTPLNPREIVTLASP
FFLNCPTGSSPAFQGFPRLNITGNLTPGTNLTIFTTNSTGATNCAFLSGLNSTFSSYSNGQC
QVPSDGKIGGGQVYVFLTNAQNVTDATTVAGPGIVEMDTPGARQPIGGMNQTLGLANVST
ANGATDGHRGASSTASTLRAASIFFASVSTAIVSFAFL

>ORY84726.1 ferritin-like domain-domain-containing protein [Leucosporidium
creatinivorum]
MVRSSLALVALAACAVQAVSIPRDEINLMKRNYIDSLSKRSKLAKRQSVTSGVGDADILNFA

LTLEHLEAAFYGQALQNFTADDFSKAGFTGVYPLLQQVSADESQHVSFLTSALSAAGATPV
EACEYTFPYTDVASFLAVSQILEGVGTSAYLGAAGAINTSAYVTAAGSILTVEARHAAFISYL
NGYSPFPAPEDTPQSAASVVTLASPFFKSCPTGSAPAIAGKPALTVTTTMPKVGSSLSIAPM
NASAVSSSGTLYCGFASGLGAGFSKWSNGTCMIPTSNVTDGQTYVTLTTGPSVSDDSVVA
GPAVIILGANNYTISAKMGSTTNSSGSASGVSSGSMPSGTSAPASGANMLQASGALAALA
GAVALLL

>XP_051412640.1 ferritin-like domain-containing protein [Gamsiella multidivaricata]
MRFTTIACALVAAATAFAAPVRKNSATSVLNYALTLEHLESEFYKQGLAKFDESSFTDAGFD
AKVRDRLVHIGEHESDHVSTLTSVIKSLKAKPVPVCEYNFPMDNITQFLAIAQALENTGVSA
YLGAASGLSGDLLTAAAQITTVEARHASYLNELWGQLGAPYSFDTALSSEQIVTIATSFIKSC
PYDLGVKPFNHLTATLPSEDSTKVTTAFTGKGANSNKTYCQFLYGNKNAVSPRSECTLPAD
AEGYVFVIVTDSKTPVTLTSQSNILAGPTLLFSGTHMH

>tr|A0A010Q9S5|A0A010Q9S5_9PEZI Rds1 OS=Colletotrichum fioriniae PJ7
MAPRSMFRLLALASGASAVPFVSEPQTTVTSEPTITPSQVAVTNVTSHGPYTGPSPTTTGA
LSTSVLASEVPILPPPDDAYDYPADGKLHGDQPAPYTPSGGIGTNGSAPVYRVQSDFDYQ
SLALALYQEYIELDLFHWGLATFSDEEFEELGLNAEDRYLLQFMAEQEIGHATVITNMLGAQ
APKQCTYNYPVTNLREYIDFNQKLTRWGESGVYGFLPHLNSGPAAQLLLQSITTEARQQMI
FRQFEGLFPMPEWHIPGIPQSWAWTLLAPYISSCPADQTRLIWQNFPALHILNQPNPYRIN
GSNVWNETTGGWANTAATTNITDAESCVNATDPLQDCNAAITQNRTMPLSYPGRQVFFQ
WDAPGQAVGPNNSYITATNVVEPKFAAWVSQLNVTFSPLMNVSLESRTAYTIQPNVSTWE
HDPAINSTMFVALTDTDMYVTAHNLTMINPHVAALAVYQAG

>sp|P53693|RDS1_SCHPO Protein rds1 OS=Schizosaccharomyces pombe (strain 972 / ATCC 24843)
MVQALTASLMAGALLARGIIGAKADPVNFAGIGGAAYEYNYTATGSFNQSIMPANFTPAGGI
DTNDSSPTYHPFSDFDYQSLSLALYHEYIEYDLFNYGLTKFSDAEFDEAGIDAEYRHLIRFM
AQQEIGHIELVTNMLGPNAPKACSYQYNFDTVGSFIDFAQTLTKWSESGVYGFLPHLDSRA
AAALLLQSITTEARQQMSLRQLQGLFPYPVWFETGIPQSFAWSLIAPFIVGCPAENEKLVW
QNFPALHLVSPPVHTNFTNGSFPQYPNGTYMYPAAVSTNRTFPLSLPGQSVELAWDAPGM
AVGPNSSYITSTSAGTPRYAAWISQLNVTYAPLNITGNNSGVTYQPSSHLYNDSTQQVINGT
NFLVLVDEAIPVTPFNITAINEHVVAGPLVYESG

>tr|A0A017SAR3|A0A017SAR3_ASPRC Protein rds1 OS=Aspergillus ruber (strain CBS 135680)

MASKHHLVLSLLGWLPSALALPNILARETAPPVVSFATSMDHTSFQGTPSVTGALNASSTL
AMTISSLSVEPSATTYPSDGKLQDPAPAPYVPAGGVGTNGTTPVYNAKSDFDFQSLALVLY
AEYIELDLFYDGLARFSEKEFTDAGLTAEDRYLIQFMAEQEIGHATLITNILGAKAPKQCQYK
YPYKTVREFVDFCQKLTRFSEAGVYGFLAHLDSREAATLLTQTITTEARQQMIFRQFEGLFP
MPVWFEVGVSQSWAWTLLAPYISSCPEGQTRLAWQNFPALHIVNQPNPDRINGSSAYNET
LTPGMNTLNSTGINDSCLKSDVIGEACNASITHNRTTPLSFPGREVLLTWETPGQLVGPNN
SYVTTTTASDPKFVMWVSQLNVTYTELTMGDNSTSGSTIQPNTTTFEGDPAVNGTMFIAIT
DSDPFVTSFNFSMVNAHVVAGPALYQAG

>KAH8124050.1 ferritin-like domain-containing protein [Trichoderma asperelloides]
MFFSQAAIAFLAAGLVAAAPVVEKRATITDSDILNYALTLEHLEDTFYHQGLANFTQADFAKA
GYDAIFYDNVQKLSIDESTHVSFLTSALKAAGATPVNACSYSFGVTDVASFLATASILEGVG
VSAYLGAAADIMSKTYLTAAGSILTVEARHSSYLRAHLKEAPFPQPFDAPLTLDEVYSLASG
FITSCPSSNPPLPVKAFPKLQLAPTTAMPVTAGATVTLLTPAEKGIQIYAAFIAVTGPTFVPAK
PVNGGFSIMLPKGFAGQTYVVLTTCKEGVSDDTTAAGPAIIEVCLFQVWLFAFNLY

>CRL19799.1 Ferritin/ribonucleotide reductase-like [Penicillium camemberti]
MKFTNVALTGAAIGLANAGPVSKRAISDADILNYALTLEHLEASFYEEGLKNYTQEDFIKAGM
KDPFYANLKEVASDEKEHVDFLTSTLKAAGASPVARCAYNFPSTDVSSFLALASVLEGVGV
SAYLGAAASIMSDTYLTAAGSILTTEARHSAYLRAAVGEVPFAQAFDNPLGLNEVYTVASPFI
ASCPSSNGALPVKAFPALTMSSMEGVMTGSQVHLMAGSGFNTSTSDVNAAFITVTGPVW
APLKSIGDGKFTVTVPKGVAGQSYVVLTQGNKQATDDNIVAGPAIVEVGKKTAMGTPSGMA
AMGMGGKKNMTMPTPSRSMAGSWSSSSATASASASPIYTGASQKLSGSVASVIAAGIFAA
AGLI

>tr|A0A081CK46|A0A081CK46_PSEA2 Ferritin/ribonucleotide reductase-like protein
OS=Pseudozyma antarctica
MLTKYAVLAAAAAAAVTAAPVVEKRAAPGQADIDTVILNYALTLEHLENAFYRDTLATYDAGA
FRAAGYPDWVRQRFVEIGGHEKAHVDFLSKALGDQATKECTYNFGITDVKTFVATSALLEG
IGNSAYLGAAQNITNVAYLTAAGSILTVEARHASWVASSVQQGDGFPAGFDTPLNFNQTYSL
AAPLITSCPDSNPALPVKAFPAATISGDVCGGKQVTISGDGVQSGQFAAFLTGLNVYYAPIG
DNGSVTVPSEVAYGRVYVVVTKSGDSVSDDNTVAGPAVVDIPLSAEKAEQIYSQQ

>tr|A0A016QLY0|A0A016QLY0_9DEIO Dessication-associated protein OS=Deinococcus
phoenicis
MSHDHNSPDQIVPDQNKPTQPTLDRRAALGTLGKLGLGAAAFGLAGSSALAVPARDIDVD

VLNFALNLEYLEAAFYLAAVGRLNELRAIGGGAEIRVPQGLDLMRGMQFKDSNVQAYMRDI
AEDEFQHVRFLHAALGKAAAPRPVLDLSAAFDAAGRAASGGKIRGFNPYANDLFFLHGAFI
FEDVGVTAYNGAATLLTNPAYLQAAAGILATEAYHAGAIRTLLFMRRQELAAAGLYVGQVVD
AISALRGKVGGGKDAGLSNAHGAVIAPTDPHGAAYPRTTREVLNIVYLAPGAHQGGFYPN
GLNGSIR

>tr|A0A023X345|A0A023X345_9ACTN Ferritin-like domain OS=Rubrobacter radiotolerans
OX=42256 GN=RradSPS_1609 PE=4 SV=1
MPKHEIQVPSAEDFVNKPRSRKQFFGALAAASLGAVGGGALLSGRATAQSSGNVDVDIAN
FALTLEYLEAEFYTRAVDSGVLSEATLPTVTNLRDHEVAHAEAIVGLLEGVGAAPVEKPEFT
FPADAFSSEAAILELAATFEPVGVGAYLGAAPLIESPDVLAAAGSIAGVEGEHVVAVNQLLG
VVPPANQAFPAALTRDEVLAAVAPFLGMDAMMDTGGQAL

>tr|A0A076HT03|A0A076HT03_9BACT Ferritin-like domain-containing protein
OS=Hymenobacter sp. APR13 OX=1356852 GN=N008_12040 PE=4 SV=1
MYLLKLLAELAATDSTQLEQSAPRRAALESLGRFSAKTLAAALPLGLAALPTQAGTTTILDS
LTLALQLERLQEALYARALAAPAGFFPADATIRASIVTMQRHQQQHITLLSDAITNSGGTVPA
QPNYDFTGSRNNTQPAFFASVFSNFDDFLRLAQLLEDAGVRAYKGQVQSLISNDFILQTAL
QIHATEARHAARIRIIRQKRAAVVKPWPSPADASITVAGKTDIVYSGENALEQLVPGYRPVPF
RLLPIGFPGTNILTQGVPEAFDEPLSTAQASTIMELFTY

>tr|Q9RZK8|Q9RZK8_DEIRA Dessication-associated protein OS=Deinococcus
radiodurans DR_B0118
MSTPVEPLLCLSSVCDTRKAMKEEMQSTRRRFLGMAGAMGAGAVLAGCANVGASEPTKT
NLDATIFNFALNLEYLEAAFYLAAVGRLNELTAAGGDASKVTLPSGVTGMGGTAVPGLTGDL
RAMMEEIADDELAHVKVIRSVLGSAAVAQPRLDLSASFLAAGSLASNGAITNFNPYANPLFF
LHGAFVFEDVGVTAYKGAARLLVGDKPGGNLENAAGILAVEAYHAGSIRTQLFMRRTEQAA
AGLTVEQVVQAISNLRDSVDGADDRDQGITANGNAGVLARDANIIPTDSNGIAFSRTPRQV
ANIVFLDTTGKAARGGFFPDGLTGDYSSILSL

>tr|A0A023X345|A0A023X345_9ACTN Ferritin-like domain OS=Rubrobacter radiotolerans
OX=42256 GN=RradSPS_1609 PE=4 SV=1
MPKHEIQVPSAEDFVNKPRSRKQFFGALAAASLGAVGGGALLSGRATAQSSGNVDVDIAN
FALTLEYLEAEFYTRAVDSGVLSEATLPTVTNLRDHEVAHAEAIVGLLEGVGAAPVEKPEFT
FPADAFSSEAAILELAATFEPVGVGAYLGAAPLIESPDVLAAAGSIAGVEGEHVVAVNQLLG
VVPPANQAFPAALTRDEVLAAVAPFLGMDAMMDTGGQAL

>MBA3337771.1 MAG: ferritin-like domain-containing protein [Chloroflexia bacterium]
MRNYHEDLVATVREATHKTTSRRGLLIGGAKLAAGGAAAMALASSPASLLGFRNAMAQTF
NGPVDVLNYALTLEHLEATFYVQGLNQFSATDFSGVGLSSTSYEYFGLIRDHEVTHVDTLIQ
VIGSLGGTPVSAAMYDFGYTDVTSFVAVAQVLENVGTGAYTGAAQFLIDNDDLLTAALTIHG
VEARHASYLNLLNGEVPFPAAFETPLTPAEVLAAAGPLIVSNMPATGTGSSL

>MBA3401579.1 MAG: ferritin-like domain-containing protein [Actinobacteria bacterium]
MSSQPVTKAEGVAASADTRAAFLRKAGLGSAALVAGGSLAGALPGAAKAHTTPTPPTDVD
MLNYALTLEHLEATFYVQGLEVFSRGDLTGAEFLGGFGGRIRSKVYDYFELIRTHELAHVNT
LQSVITSLGGTPVPACTYNFERTAFTSVEQFVSVARDLENTGVMAYDGAIAHIEAAALLTAG
ATIATVEARHASYLNLINGDVPFPNEFDTPVAPRVICRLVDERFIDSCPSSFDLAEFCSLLPD
TVTPTP

>RDK12355.1 DUF4439 domain-containing protein [Cupriavidus lacunae]
MITAPDARRRGLLKVPGLFALGSLAVLSLGESMPAWAQTQSGSTKDDINILNTALGLEYQAI
AAYQVGAESGLLQKPVLATAVKFQDHHKAHAQVLAGTVQKLGGSPVMAKKPSEYAFPTEQ
LKNQADVLRFAAGLEKGATAAYLGVLPNFHNRELTRAAGSILGDEAMHWAILLQVLGEDPV
PGAFVG
